# Multi-omics reveals that alkaline mineral water improves the respiratory health and growth performance of transported calves

**DOI:** 10.1186/s40168-023-01742-4

**Published:** 2024-03-08

**Authors:** Jiancheng Qi, Linli Gan, Fangyuan Huang, Yue Xie, Hongrui Guo, Hengmin Cui, Junliang Deng, Liping Gou, Dongjie Cai, Chunhui Pan, Xia Lu, Ali Mujtaba Shah, Jing Fang, Zhicai Zuo

**Affiliations:** 1 Key Laboratory of Animal Disease and Human Health of Sichuan Province, College of Veterinary Medicine, Sichuan Agricultural University, 211 Huimin Road, Wenjiang District, Chengdu, 611130 Sichuan China; 2 Sichuan Hannover Biological Technology Co. Ltd, Deyang, 618000 Sichuan China; 3 Beijing Jnnail Biological Technology Co. Ltd, Daxing, Beijing, 102600 China; 4 Key Laboratory of Animal Genetics, Breeding and Reproduction of Shaanxi Province, College of Animal Science and Technology, Northwest A&F University, Yangling, 712100 Shaanxi China

**Keywords:** Calf, Transportation stress, Alkaline mineral water, Clinical scoring system, Multi-omics

## Abstract

**Background:**

Long-distance transportation, a frequent practice in the cattle industry, stresses calves and results in morbidity, mortality, and growth suppression, leading to welfare concerns and economic losses. Alkaline mineral water (AMW) is an electrolyte additive containing multiple mineral elements and shows stress-mitigating effects on humans and bovines.

**Results:**

Here, we monitored the respiratory health status and growth performance of 60 Simmental calves subjected to 30 hours of road transportation using a clinical scoring system. Within the three days of commingling before the transportation and 30 days after the transportation, calves in the AMW group (*n* = 30) were supplied with AMW, while calves in the Control group (*n* = 29) were not. On three specific days, namely the day before transportation (day -3), the 30^th^ day (day 30), and the 60^th^ day (day 60) after transportation, sets of venous blood, serum, and nasopharyngeal swab samples were collected from 20 calves (10 from each group) for routine blood testing, whole blood transcriptomic sequencing, serology detection, serum untargeted metabolic sequencing, and 16S rRNA gene sequencing. The field data showed that calves in the AMW group displayed lower rectal temperatures (38.967 ℃ vs. 39.022 ℃; *p* = 0.004), respiratory scores (0.079 vs. 0.144; *p* < 0.001), appetite scores (0.024 vs. 0.055; *p* < 0.001), ocular and ear scores (0.185 vs. 0.338; *p* < 0.001), nasal discharge scores (0.143 vs. 0.241; *p* < 0.001), and higher body weight gains (30.870 kg vs. 7.552 kg; *p* < 0.001). The outcomes of laboratory and high throughput sequencing data revealed that the calves in the AMW group demonstrated higher cellular and humoral immunities, antioxidant capacities, lower inflammatory levels, and intestinal absorption and lipogenesis on days -3 and 60. The nasopharynx 16S rRNA gene microbiome analysis revealed the different composition and structure of the nasopharyngeal microflora in the two groups of calves on day 30. Joint analysis of multi-omics revealed that on days -3 and 30, bile secretion was a shared pathway enriched by differentially expressed genes and metabolites, and there were strong correlations between the differentially expressed metabolites and the main genera in the nasopharynx.

**Conclusions:**

These results suggest that AMW supplementation enhances peripheral immunity, nutrition absorption, and metabolic processes, subsequently affecting the nasopharyngeal microbiota and improving the respiratory health and growth performance of transported calves. This investigation provided a practical approach to mitigate transportation stress and explored its underlying mechanisms, which are beneficial for the development of the livestock industry.

Video Abstract

**Supplementary Information:**

The online version contains supplementary material available at 10.1186/s40168-023-01742-4.

## Introduction

In the cattle industry, transportation is a routine procedure that can profoundly impact the animals’ well-being [1]. Cattle will be subject to various stressors during this process, including exposure to different handling techniques, mingling with unfamiliar individuals in new environments, being deprived of food and water, and suffering from fluctuating temperatures, making transportation a highly stressful event for them [2, 3]. While transportation is a known source of stress for cattle of all ages, it poses a particularly high risk to young calves. The fasting and dehydration accompanying the process can leave them susceptible to energy depletion, hunger, and hypoglycemia, as they lack the body fat reserves compared to adult cattle [1, 4]. Primarily, the intermixing of calves at auction markets, during transportation, and in lairage can heighten their exposure to pathogens and result in social stress that can impede immunity and pathogen excretion [5, 6]. It was reported that 12% of the calves at an auction market, 42% of the calves on arrival at their destination, and 50% of the calves in the first week after arrival at the facility showed signs of illness [7].

The primary concern for young calves is infectious diseases, which pose a significant challenge for calf raisers following transportation. In addition, the likelihood of it intensifying the transportation pressure and deprivation during transit is high [1]. Indeed, transport has been established as a crucial element contributing to the development and progression of bovine respiratory disease (BRD) [8, 9]. For now, BRD is considered one of the most prevalent causes of pneumonia in cattle worldwide and results in morbidity and mortality in newly transported calves, leading to a slower growth rate and economic consequences [10]. As a result, one of the utmost priorities in the cattle industry should be to minimize stress levels during and after transportation.

Optimizing pre- and post-transport calf management, such as proper colostrum management, nutrition, housing, hygiene, and supplementation with additive agents, is a recognized approach to minimizing stress levels [1]. For example, supplementation of 10% of molasses-based liquid, blood serum-derived proteins, and fructooligosaccharides were found to reduce the morbidity and mortality of transported calves and increase their growth performance [11, 12]. However, the underlying mechanisms are still unclear, probably because conducting experimental research on live calves is expensive and inconvenient. Extensive research and studies have been conducted on the health and medical benefits of alkaline mineral water (AMW), which contains essential elements such as Na, K, Zn, metasilicic acid, and rare minerals like germanium [13], which are critical for organ function (heart, brain, and gut), as well as various physiological function such as digestion and immunological biosynthesis [14]. These studies have been conducted in human medicine and have shown significant improvements in the quality of life of cancer patients, antioxidant effects, promotion of intestinal health, and treatment of intestinal inflammatory diseases and diarrhea [15, 16]. Additionally, the animal industry has benefited from using AMW in mitigating heat-induced stress and improving growth performance in cattle [16], improving intestinal barrier function and alleviating diarrhea in cattle and piglets [17]. These outcomes suggest that AMW supplementing might enhance the respiratory tract health and growth performance of transported calves. Nevertheless, this supposition needs to be validated.

The respiratory tract plays a crucial role in the respiratory system and hosts diverse microbial communities in distinct ecological niches, including the nasopharynx [18]. The microbiota residing in the latter can offer an all-encompassing understanding of the microbiota throughout the respiratory tract [19]. And the composition and structure of calf nasopharyngeal microbiota have been acknowledged as important indicators of the general well-being of the bovine respiratory system [10, 20]. The establishment of nasopharyngeal microflora is a complex phenomenon impacted by many variables, such as the surrounding environment and the host’s immune response [20].

However, it remains uncertain whether the supplementation of AMW would provide benefits in reducing transportation stress, improving the respiratory health status, and enhancing growth performance of transported calves. Thus, we monitored the respiratory well-being and growth performance of calves transported over long distances and supplied with AMW compared to a group of control calves. To analyze the nasopharyngeal microflora, immune system responses, and metabolic processes of the transported calves before, 30 days, and 60 days after transportation, we utilized 16S rRNA gene microbiome sequencing, whole blood transcriptome sequencing, and serum untargeted metabolism sequencing techniques. These results were validated via serological detection and routine blood test (RBT). This study will validate the benefits of AMW in lowering the stress levels of transported calves, disclosing its underlying mechanisms, and contributing to the progress of the cattle industry.

## Methods

All animal-related activities and procedures were conducted under the watchful eye of the veterinarians affiliated with Caijiashan Breeding Company, a large-scale beef cattle breeding establishment in Quxian County, Dazhou City, Sichuan Province, China.

### Animals’ management

The administrator of Caijiashan Breeding Company purchased 116 Simmental crossbred calves, approximately eight months old, in excellent health (without any signs of illness) from nearby farms in Yitong County, Siping City, Jilin Province, China. Each of the 116 calves was randomly assigned a unique number from 1 to 116. Subsequently, they were randomly divided into two adjoining pens, namely pen I and pen II. Pen I housed 63 calves, while pen II accommodated 53 calves. The objective of this configuration was to oversee their behavior and facilitate their mingling for three consecutive days. Twice daily, at 8:30 am and 4:30 pm, the calves were supplied with hay, concentrate, and water, with a minimum of 5-10% of the fodder remaining after each feeding. The health status of all calves was subject to constant scrutiny by a veterinarian employed by the company. After a thorough examination, it was observed that a calf, Number 29 in pen I, exhibited symptoms indicative of respiratory distress, resulting in its return to its former farmer. Once the commingling was complete, the remaining 115 calves (62 from Pen I and 53 from Pen II) were transferred to a spacious truck covered with hay. The truck was then used to transport the calves to the company’s feedlot via the highway. The truck’s roof was enclosed, and the distance travelled was approximately 3,000 km. During the 30-hour transit, the calves were not supplied with food or water. Upon arrival, the calves were unloaded and distributed into two nearby enclosures. The 62 calves on the upper floor were assigned to pen A (80.0 × 10.0 m; *n* = 62), and the 53 calves on the lower floor were assigned to pen B (80.0 × 10.0 m; *n* = 53). Following this, all calves were tethered to the railings at 2.0 m intervals, then were given three liters (L) of brown sugar and ginger (BSG) water (0.5 kg/10 L and 0.3 kg/10 L, respectively) and were prevented from accessing food for six hours. Then, they were provided with the total mixed ration (TMR) that included forage, silage, and hay. The composition and nutrient levels of the TMR are outlined in Supplementary Table 1. During the following six days of adaptive feeding, the calves were allowed to drink regular water and consume TMR (with 5-10% leftovers) twice daily after consuming the BSG water. After the adaptive feeding, the calves were allowed to access normal water and TMR freely. All treatments were determined and administered solely by the staff at the feedlot based on their expertise.

### Experimental design

**Fig. 1:**
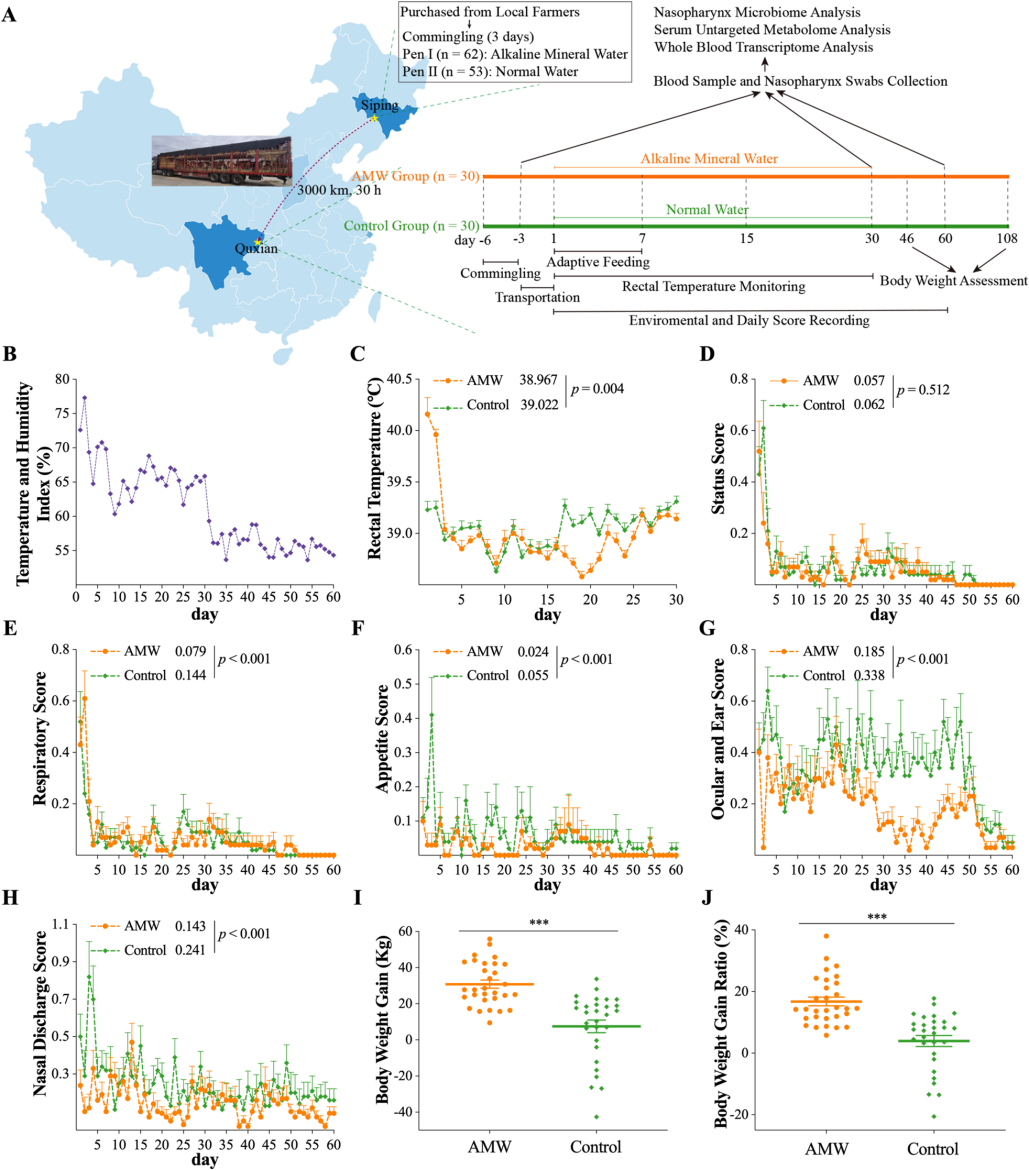
A Sketch Map of the Experimental Design and the Physiological Manifestations of Calves. **A**, the sketch map of the experimental design and procedures in this study; **B**, the temperature and humidity indexes (THIs) during the 60 days after the arrival of the calves; **C**, the rectal temperatures of each experimental calf during the 30 days after their arrival; **D-H**, the status scores, respiratory scores, appetite scores, ocular and ear scores, and nasal discharge scores of the experimental calves during the 60 days after their arrival; **I**, the increased body weight of experimental calves from day 46 to day 108; and **J**, the ratio of the increased body weight of calves from day 46 to day 108 to their initial body weight on day 46. The data are expressed as the mean ± the standard error mean (SEM). In **C**-**H**, a generalized linear mixed model was used to analyze the differences in the rectal temperature and the daily scores between the two groups. For **I** and **J**, a student t-test (**I**) and a Mann-Whitney U test (**J**) were used to discern the differences in the body weight gain between the two groups: ***, *p* < 0.001

For the three days of commingling, a solution of AMW stoste (Beijing Jnnail Biological Technology Co., Ltd, Beijing, China. Cat No: Q/NEL 005-2017) was provided to the calves in pen I, at a dosage of 30 ml per calf per day, blended in their drinking water. This treatment method and dosage were determined based on the recommendation of its manufacturer. As the commingling was about to end (day -3), ten calves from Pen I and ten from Pen II were randomly selected, marked, and had their blood samples and nasopharyngeal swabs collected. Upon their arrival, two groups, namely the AMW group and Control group, were set up for the experiment. The AMW group comprised the ten marked calves in pen A and an additional 20 randomly selected calves in pen A. Meanwhile, the Control group comprised the ten marked calves in pen B and 20 randomly selected calves from pen B. The calves in the AMW group were administered AMW stoste with a dosage of 30 ml per calf per day for 30 days after arriving at the feedlot, while the calves in the Control group were not given AMW stoste and served as the control. We monitored the calves for 108 days after their arrival at the feedlot, and Fig. 1A illustrates the experimental design.

### Measurement of Temperature and Humidity Indexes (THIs)

To investigate the potential impact of environmental factors, such as THIs, on our outcomes, we collected data on daily temperatures and relative humidity in the pens for 60 consecutive days from their arrival. We used a Deli 9010 In-Outdoor Thermo-Hygrometer from Deli Group Co., Ltd. (Beijing, China) to measure the environmental temperature (T, ℃) and relative humidity (RH, %) in the feedlot for 60 consecutive days at 8:00 am and 3:00 pm every day. The THI for each day was calculated using the following formula [21]:

$${\text{THI}}=\left(1.8\times {\text{T}}+32\right)-\left(0.55-0.0055\times {\text{RH}}\right)\times \left(1.8\times -26\right)$$

### Detection of rectal temperature

Our previous study found that the rectal temperature is a simple and sensitive indicator of calf welfare [21]. Therefore, we assessed the rectal temperature (℃) of each calf in the AMW and Control groups using a soft-head electronic thermometer (BT-A21G, Fudakang Industry Co., Ltd., Guangdong, China) at 7:00 am and 2:30 pm each day from day 1 to day 30. The average values of the two readings of a day were used to determine the rectal temperature of that particular day.

### Evaluation of BRD-related daily scores

Building on a previous clinical scoring system [22], we developed a clinical scoring system better suited to local beef cattle. This system evaluates the status, respiration, appetite, ocular and ear and nasal discharge scores. The quantitative criteria for each score are outlined in Table 1. All the daily scores of calves in the AMW and Control groups from day 1 to 60 were assessed by a single veterinarian who was qualified to do so. The average values of the two recordings of a day were used to determine the scores of that particular day.

**Table 1:** The quantitative criteria of the clinical scoring system used in this study

**items**	**criterion**	**score**
**status**	regular activity, prompt alertness, and swift reaction	0
	slightly reduced activity, incomplete alertness, and mild depression	1
	more often lying down, slower response, and depressed demeanor	2
	long time lying down, barely respond to stimulation, and cannot stand up	3
**respiratory**	regular respiratory rate (10~30 times/min), and no cough	0
	slightly increased respiratory rate (31~45 times/min) and/or occasional coughs	1
	a moderate increase in respiratory rate (46~55 times/min) and/or noticeable cough (multiple coughs within 5 minutes)	2
	dyspnea (more than 55 times/min), wheezing, and open-mouth breathing	3
**appetite**	quickly eat all the forage	0
	eat 4/5 of the forage or slowly eat all the forage	1
	eat 3/5 of the forage	2
	eat 1/5 of the forage or less	3
**ocular and ear**	normal	0
	slight ocular secretion and ear bounce	1
	moderate bilateral ocular secretion or mild unilateral earlobe	2
	severe eye secretion, severe head tilt, or bilateral ear droop	3
**nasal discharge**	no nasal discharge	0
	the small amount of serous nasal discharge	1
	flowing mucus or yellow viscous nasal discharge	2
	mucosal cyanosis, and/or purulent or rusty nasal discharge	3

### Assessment of body weight

Utilizing a weighbridge, the body weight of each calf in the AMW and Control groups was measured on days 46 and 108.

### Nasopharynx swab collection, 16S rRNA gene sequencing, and microbiome analysis

On days -3, 30, and 60, 20-cm sterile deep nasopharyngeal swabs (Merlin Technology, Tianjing, China) were employed to take samples of the nasopharyngeal microflora from the mucosa of the 20 marked calves. The swabs were quickly stored in dry ice and dispatched to Novogene Biotech Co., Ltd. (Beijing, China) within three days for microbiome sequencing and analysis. Briefly, the total genomic DNA from swabs was extracted using the hexadecyltrimethylammonium bromide (CATB) method to obtain DNA from certain bacteria which possess resilient cell walls, and the quality and integrity of each DNA sample were assessed by electrophoresis in a 1% agarose gel with Tris-acetate-Ethylene Diamine Tetraacetic Acid (EDTA) buffer. The concentration of the DNA was determined using the NanoDrop ND-2000 spectrophotometer (Thermo Fisher, Waltham, MA, USA). Subsequently, 60 libraries were constructed and sequenced using the Illumina MiSeq sequencing platform. Polymerase chain reaction (PCR) amplifications were conducted from each sample to generate the V3-V4 hypervariable region (341F: 5’-CCTAYGGGRBGCASCAG-3’, 806R: 5’-GGACTACNNGGGTATCTAAT-3’) of the 16S rRNA gene, following previously described methods [23]. Following the removal of barcode and primer sequences, the reads of each sample were merged using FLASH V 1.2.7 (http://ccb.jhu.edu/software/FLASH/index.shtml) [24], and quality filtering of the raw tags were conducted through QIIME (v 1.9.1, http://qiime.org/scripts/split_libraries_fastq.html). Subsequently, the sequences were clustered into operational taxonomic units (OTUs) with 97% identity, with the most frequent OTU sequence chosen as the representative OTU sequence and annotated. After homogenizing the sample data, QIIME was used to calculate the α and β diversity. The ade4 and vegan packages in R software (v2.15.3) were employed to generate principal coordinate analysis (PCoA) diagrams and perform similarity percentage (SIMPER) [25] analysis. The nonparametric test was applied to analyze the difference in microbiota. Finally, the PICRUSt method [26] was used to predict the functions of OTUs.

### Blood sample collection and processing

On days -3, 30, and 60, a 20 ml blood sample was taken from the right jugular vein of the 20 marked calves in pens A and B. Three drops (about 0.2 mL) of the blood sample were transferred to an EDTA anticoagulant tube for an RBT using an automated hemocytometer (Pukang automatic animal blood cell analyzer PE-6800VET, Pukang Electronic Co., Ltd, Shenzhen, China) within two hours. Additionally, 15 mL of the blood was transferred to a sodium-heparinized, non-anticoagulant tube and left to stand for three hours. Afterwards, the blood was centrifuged at 1500 × g for 10 minutes to collect the serum, 300 μL of which was stored in dry ice and sent to Novogene Biotech Co., Ltd. within three days for Liquid Chromatography-Mass Spectrometry (LC-MS) untargeted metabolome sequencing. The remaining serum was stored at -80 ℃ for further laboratory experiments. The remaining five mL of blood was transferred to an EDTA anticoagulant vacuum tube. The blood and anticoagulant were mixed and placed in a frozen storage tube. After that, three times the volume of Trizol reagent (Transgen Biotech, Beijing, China; Cat No: ET111-01) was added to the storage tube, and the mixture was shaken thoroughly. The blood samples were finally stored in dry ice and sent to Novogene Biotech Co., Ltd. within three days for transcriptome sequencing and analysis.

### Untargeted metabolomics by LC-MS and metabolomic analysis

The untargeted metabolomic sequencing and analysis were carried by the Novogene Co., Ltd. The process started with the resuspension of 100 μL of serum in 400 μL of pre-chilled 80% methanol and 0.1% formic acid, through vortexing. The mixture was then incubated on ice for five minutes and centrifuged at 15000 × g for 20 minutes at 4 ℃. The resulting supernatant was diluted to a final concentration of 53% methanol using LC-MS grade water and centrifuged again under the same conditions. The supernatant was introduced into an LC-MS/MS system, consisting of a Vanquish UHPLC system (Thermo Fisher, Waltham, MA, USA) coupled with an Orbitrap Q ExactiveTMHF-X mass spectrometer (Thermo Fisher, Waltham, MA, USA) for analysis. The raw data obtained from the UHPLC-MS/MS were processed using Compound Discoverer 3.1 (CD3.1, Thermo Fisher, Waltham, MA, USA). This software facilitated peak alignment, peak picking, and quantification for each metabolite. The metabolites were then annotated using the Kyoto Encyclopedia of Genes and Genomes (KEGG) database (https://www.genome.jp/kegg/pathway.html) and the Lipid Maps database (http://www.lipidmaps.org/). Visual representations of PCA were generated using metaX software, and the functions of metabolites and metabolic pathways were analyzed using MetaboAnalyst 5.0 (https://www.metaboanalyst.ca/).

### Whole blood transcriptome sequencing and transcriptomic analysis

The transcriptome sequencing and analysis were performed by Novogene Co., Ltd. In brief, total RNA was extracted using a commercialized kit (TransGen, Beijing, China; Cat No: ET111-01) employing the traditional phenol/chloroform phase separation method. The concentration of the extracted RNA was measured using a NanoDrop ND-2000 Spectrophotometer (Thermo Scientific, Wilmington, USA). The integrity of the extracted RNA was assessed using the Bioanalyzer 2100 system’s RNA Nano 6000 Assay Kit (Agilent Technologies, CA, USA). Only samples with an RNA integrity number (RIN) greater than 0.8 were selected for sequencing. Complementary DNA (cDNA) libraries were constructed using an Illumina TruSeq RNA sample prep kit (Illumina, San Diego, CA, USA), resulting in an average size of 150 bp (excluding adaptors). The quality and integrity of the libraries were assessed using the Agilent 2100 Bioanalyzer and the ABI StepOne Plus real-time PCR system. The results indicated that all samples fulfilled the requirements for library selection. Firstly, they exhibited narrow distributions with a peak size of around 275 bp. Secondly, they possessed an effective concentration of at least 1.5 nM. The RNA-Seq FASTQ files were aligned with the bovine genome (http://ftp.ensembl.org/pub/release-105/fasta/bos_taurus/) using the Hisat2 algorithm [27]. The resulting binary alignment/map (BAM) format output files were processed by Cufflinks to assess transcript abundance and detect potential mRNA isoforms. StringTie (v1.3.3b) was utilized to assemble the mapped reads of each sample based on a reference approach. The expression levels of transcripts were measured using the fragments per kilobase million (FPKM). Principle Component Analysis (PCA) was also performed based on FPKM values. To identify differentially expressed genes (DEGs) between groups at a specific time point, a set of criteria was defined: |log2(FoldChange)| ≥ 1 and *p* ≤ 0.05. The KEGG enrichment analysis was performed using the ClusterProfiler R package.

### Serology detection

The liver function indicators, including total protein (TP), albumin (ALB), globulin (GLB), the ratio of ALB to GLB (A/G), alanine aminotransferase (ALT), aspartate aminotransferase (AST), the ratio of AST to ALT, alkaline phosphatase (ALP), gamma-glutamyltransferase (GGT), total bilirubin (TBIL), direct bilirubin (DBIL), and indirect bilirubin (IBIL); the renal function indicators, including creatinine (CRE) and blood urea nitrogen (BUN); the glucose metabolism indicator, glucose (GLU); the lipid metabolism indicators, including total cholesterol (TC), triglycerides (TG), high-density lipoprotein cholesterol (HDL-C), low-density lipoprotein cholesterol (LDL-C), and very low-density lipoprotein cholesterol (VLDL-C); and the activities of the myocardial enzymes, including lactate dehydrogenase (LDH) and creatine kinase (CK), were assessed using the Hitachi 7180 automatic biochemical instrument (Olympus Corporation, Japan). Various immunology-related indicators were measured using commercial enzyme-linked immunosorbent assay (ELISA) kits. These indicators included immunoglobulin A (IgA; # H108-1-2), IgG (# H106-1-2), IgM (# H109-1-2), interleukins (ILs)-2 (# H003-1-2), 6 (# H007-1-2), 8 (# H008-1-2), and 10 (# H009-1-2), interferon (IFN)-γ (# H025-1-2), tumor necrosis factor (TNF)-α (# H052-1-2), serum amyloid A (SAA; # H134), C-reactive protein (CRP; # H126-1-2), and heat shock 70 kDa protein (HSP70; # H264-2-2). Additionally, antioxidant indicators such as hydroxyl radical (·OH; # A018-1-1), nitric oxide (NO; # A012-1-2), and malondialdehyde (MDA; # A003-1-2) were measured. The activities of antioxidant enzymes including total antioxidant capacity (T-AOC; # A015-2-1), superoxide dismutase (SOD; # A001-3-2), glutathione peroxidase (GSH-Px; # A005-1-2), catalase (CAT; # A007-1-1), and nitric oxide synthase (NOS; # A014-2-2) were also assessed. Furthermore, serum hormone levels of adrenocorticotropic hormone (ACTH; # H097-1-2), cortisol (COR; # H094-1-2), growth hormone (GH; # H091-1-2), and antidiuretic hormone (ADH; # H396-1) were measured. Enzyme activities related to glucose metabolism, such as glucose-6-phosphate dehydrogenase (G-6-PDH; # A027-1-1), pyruvate kinase (PK; # A076-1-1), and phosphofructose kinase (PFK; # H244), were determined. Lastly, the serum levels of lipid metabolism indicators, including non-esterified fatty acids (NEFAs; # A042-2-1), fatty acid synthetase (FAS; # H231-1-2), and acetyl CoA carboxylase (ACC; # H232-1-2) were measured. All these ELISA kits were purchased from Nanjing Jiancheng Bioengineering Institute (Nanjing, China) and their catalog number were listed in the brackets.

### Statistical analysis

The results of the study were presented as mean ± standard deviation (SD) and analyzed using SPSS 26 software (IBM, NYC, USA). Graphs were created using GraphPad Prism 9.3 (GraphPad Software, CA, USA), Adobe Illustrator (Adobe Systems Incorporated, CA, USA), or Novomagic (http://magic.novogene.com) unless otherwise stated. The rectal temperature and daily score data were analyzed using Generalized Linear Mixed Models (GLMM), with temperature or scores as the target, “group” and “THI” as the fixed effects, and the “day” as repeat measurement. Meanwhile, the body weight data, as well as the RBT and serum indicators were analyzed using either a t-test/one-way analysis of variance analysis (for parametric data) or the Mann-Whitney U test/Wilcoxon Rank Sum test (for nonparametric data). A *p*-value < 0.05 was considered statistically significant. The metabolites with variable importance in the projection (VIP) > 1, *p* < 0.05, and |log2(FoldChange)| ≥ 1| were identified as differentially expressed metabolites (DE Meta). Differential expression analysis was accomplished using the DESeq2 R package (1.20.0). Benjamini and Hochberg’s approach was employed to control the false discovery rate, and genes with an adjusted *p* ≤ 0.05 were identified as differentially expressed. For KEGG enrichment analysis, terms with an adjusted *p*-value of ≤ 0.05 were deemed to be significantly enriched by DEGs.

## Results

Within the first two months, an experimental calf (Number 78) from the Control group passed away. This calf was not among the 20 marked calves and was not considered in the experiment. As a result, the AMW groups consisted of 30 calves, while the Control group comprised 29. The THIs experienced a swift decline from the initial day to the 60^th^ day in the feedlot (Fig. 1B).

### Physiological manifestations of calves

Calves in the Control group had a significantly higher rectal temperature (39.022 ℃) than those in the AMW group (38.967 ℃, *p* = 0.004) (Fig. 1C). The status scores of calves in the two groups were similar (*p* = 0.512, Fig. 1D). The respiratory scores, appetite scores, ocular and ear scores, and nasal discharge scores of the calves in the AMW group were significantly lower than those in the Control group (*p* < 0.001; Fig. 1E-H). The rectal temperature and all the five daily scores significantly correlate with the THIs (*p* < 0.001; Supplementary Figure 1A-F). During the period from day 46 to day 108, calves in the AMW group experienced a significantly higher increase in body weight (30.87 kg) compared to those in the Control group (7.552 kg, *p* < 0.001; Fig. 1I). Moreover, the ratio of the increased body weight of each experimental calf to its body weight on day 46 showed similar results with the body weight gain (Fig. 1J).

### Nasopharyngeal microbiome profiles of calves

**Fig. 2:**
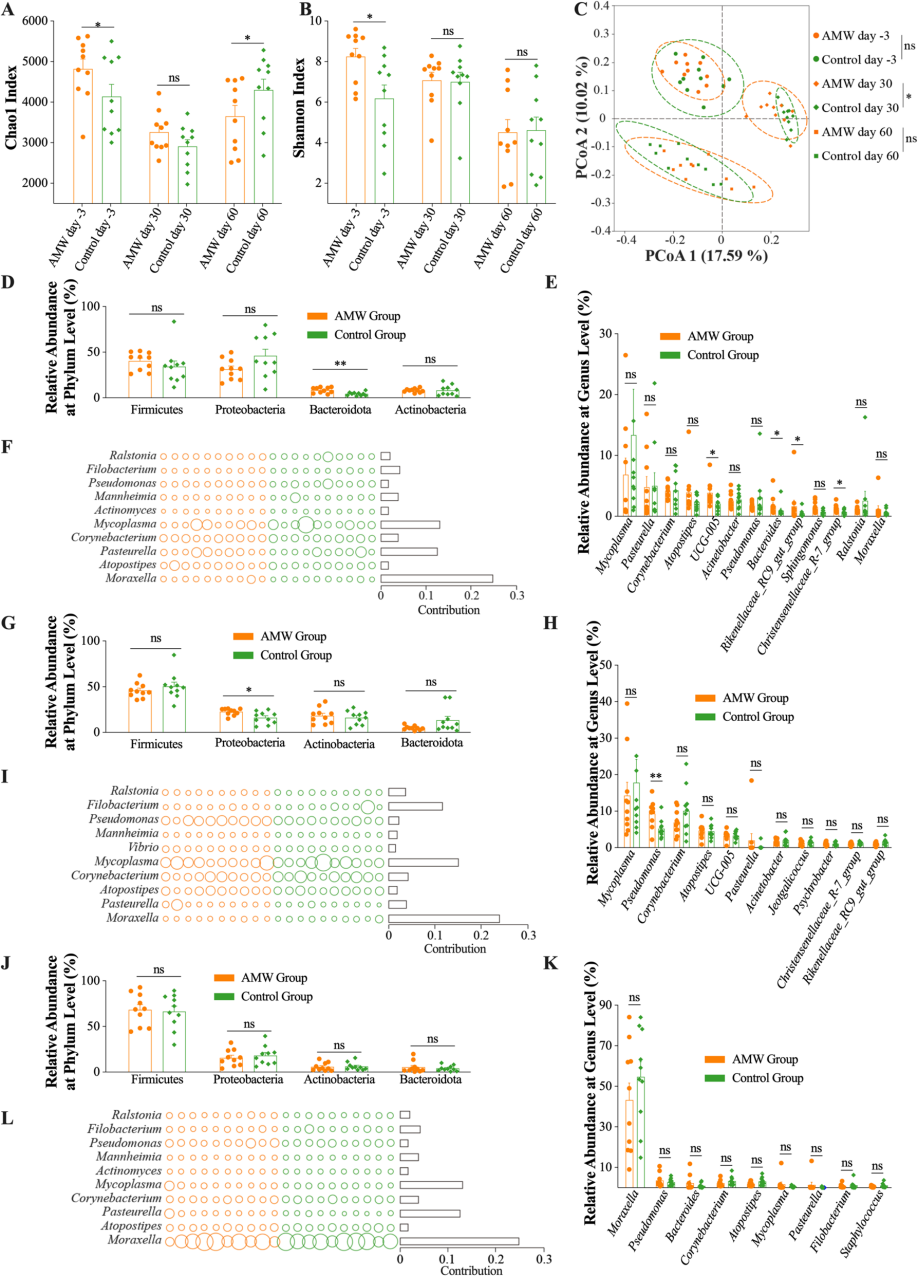
The Structure and Composition of the Nasopharyngeal Microbiota in Calves. **A** and **B**, the Chao1 and Shannon indexes of the nasopharyngeal microflora in calves; **C**, the results of the Unweighted Unifrac-based Principal Coordinates Analysis (PCoA) of nasopharyngeal microflora in calves; and **D-K**, the relative abundance of phyla (**D**, **G**, and **J**) and genera (**E**, **H**, and **K**) with > 1% relative abundance, and the genera with the top 10 contributions to the difference between the two groups (**F**, **I**, and **L**), in the nasopharyngeal microbiota on days -3, 30, and 60, respectively. The data are expressed as the mean ± standard error mean (SEM). In **A** and **B**, the least significance difference method in the one-way analysis of variance was used to analyze the differences between the AMW and Control groups at each time point: ns, *p* > 0.05; *, *p* < 0.05. In **C**, the permutational multivariate analysis of variance was used to calculate the difference between the AMW and Control groups at each time point: ns, *p* > 0.05; *, *p* < 0.05. In **D**-**K**, the Wilcox Rank-Sum test was used to analyze the difference in microbiota between the two groups at each time point: ns, *p* > 0.05; *, *p* < 0.05; **, *p* < 0.01

The 16S rRNA gene sequencing process yielded a total of 5,035,998 raw reads, with an average of 83,933 ± 8,524 reads per sample (Supplementary Table 2). After quality checking, the sequences were clustered into 17,687 OUTs at 97% sequence similarity with an average good coverage of 98.27 ± 0.45% in all the samples. The rarefaction curves of the microbiota samples collected from the 20 marked calves in the two groups at three different time points tended to flatten (Supplementary Figure 2A). On day -3, the total number of observed species in the nasopharynx of calves in the AMW group were significantly greater than those in the Control group (*p* < 0.05). However, on days 30 and 60, the numbers of species observed in the two groups were similar (*p* > 0.05; Supplementary Figure 2B). The nasopharyngeal microbiota community of calves in the AMW group exhibited marked increases in Chao1 and Shannon indices, which are indicative of α diversity in the microbiota community, compared to the Control group (*p* < 0.05) on day -3. However, on day 30, the two groups had no significant difference in these indices (*p* > 0.05). On day 60, the Chao1 index of the nasopharyngeal microbiota community of calves in the AMW group was significantly lower (*p* < 0.05), and the Shannon index was similar (*p* > 0.05) to that of the Control group (Fig. 2A and B). The nasopharyngeal microbiota of calves in the AMW and Control groups on days -3, 30, and 60 underwent a PCoA based on the Unweighted Unifrac, and the results are shown in Fig. 2C. According to the results of the analysis of similarity (ANOSIM), the nasal microbiota of calves in the AMW groups exhibited similarity to those in the Control group on days -3 and 60 (*p* > 0.05). However, this similarity was not observed on day 30 (*p* < 0.05).

Supplementary Table 3 provides a detailed description of the annotation details of the OTUs, as well as the statistical variances in microbiota between the AMW and Control groups at each time point. Specifically, Proteobacteria, Firmicutes, Bacteroidota, and Actinobacteria were the most abundant phyla. Among the phyla with a relative abundance greater than 1%, Bacteroidota (*p* < 0.01) and Proteobacteria (*p* < 0.05) exhibited higher relative abundances on the nasopharynx of calves in the AMW group than those in the Control group on day -3 and 30 (Fig. 2D and G), respectively. In contrast, the relative abundances of all these phyla were similar on the nasopharynx of calves in the AMW and Control groups (*p* > 0.05) on day 60 (Fig. 2J). At the genus level, *Moraxella*, *Mycoplasma*, *Pasteurella*, *Filobacterium*, *Corynebacterium*, *Mannheimia*, *Ralstonia*, *Pseudomonas*, and *Atopositipes* were abundant in the nasopharynx of calves (Supplementary Figure 2C). We focused on the genus with relative abundances greater than 1%. On day -3, it was observed that the nasopharynx of calves in the AMW group had higher (*p* < 0.05) relative abundances of *UCG-005*, *Bacteroides*, *Rikenellaceae RC9 gut group*, and *Christensenellaceae R-7 group* compared to those in the Control group. However, the other nine genera were found to have comparable levels between the two groups (*p* > 0.05; Fig. 2E). The SIMPER analysis revealed that *Moraxella*, *Mycoplasma*, and *Pasteurella* mainly contributed (> 0.1) to the difference between the nasopharyngeal microbiota of calves in the AMW and Control groups on day -3 (Fig. 2F and Supplementary Table 4). On day 30, the *Pseudomonas* was more abundant in the nasopharynx of calves in the AMW group than those in the Control group (*p* < 0.05), while the other ten genera remained comparable (*p* > 0.05; Fig. 2H). And *Moraxella*, *Mycoplasma*, and *Filobacterium* mainly contributed (> 0.1) to the difference in the nasopharyngeal microbiota community of calves between the AMW and Control groups (Fig. 2I and Supplementary Table 4). On day 60, the relative abundances of the genera with > 1% relative abundance were comparable between the nasopharyngeal microbiota of calves in the AMW and Control groups (*p* > 0.05), and the *Moraxella*, *Mycoplasma*, and *Pasteurella* were the most contributing genera (Fig. 2K and L and Supplementary Table 4). At the species level, the relative abundances of the four species associated with BRD, namely *Pasteurella multocida*, *Mannheimia haemolytica, Histophilus somni*, and *Mycoplasmopsis bovis*, were found to be abundant in the nasopharyngeal microbiota on day -3 (Supplementary Figure 2D). On day -3, *Histophilus somni* was more abundant (*p* < 0.05) in the nasopharynx of calves in the Control group than those in the AMW group (Supplementary Figure 2H). However, there was no difference in the abundance of these four species between groups on the three days (*p* > 0.05; Supplementary Figure 2E-H). Finally, there was no significant difference (*p* > 0.05) in the PICRUSt method predicted functions of the microorganisms present in the nasopharynx of the calves (Supplementary Figure 2I).

### Transcriptome profiles of white blood cells from calves

**Fig. 3:**
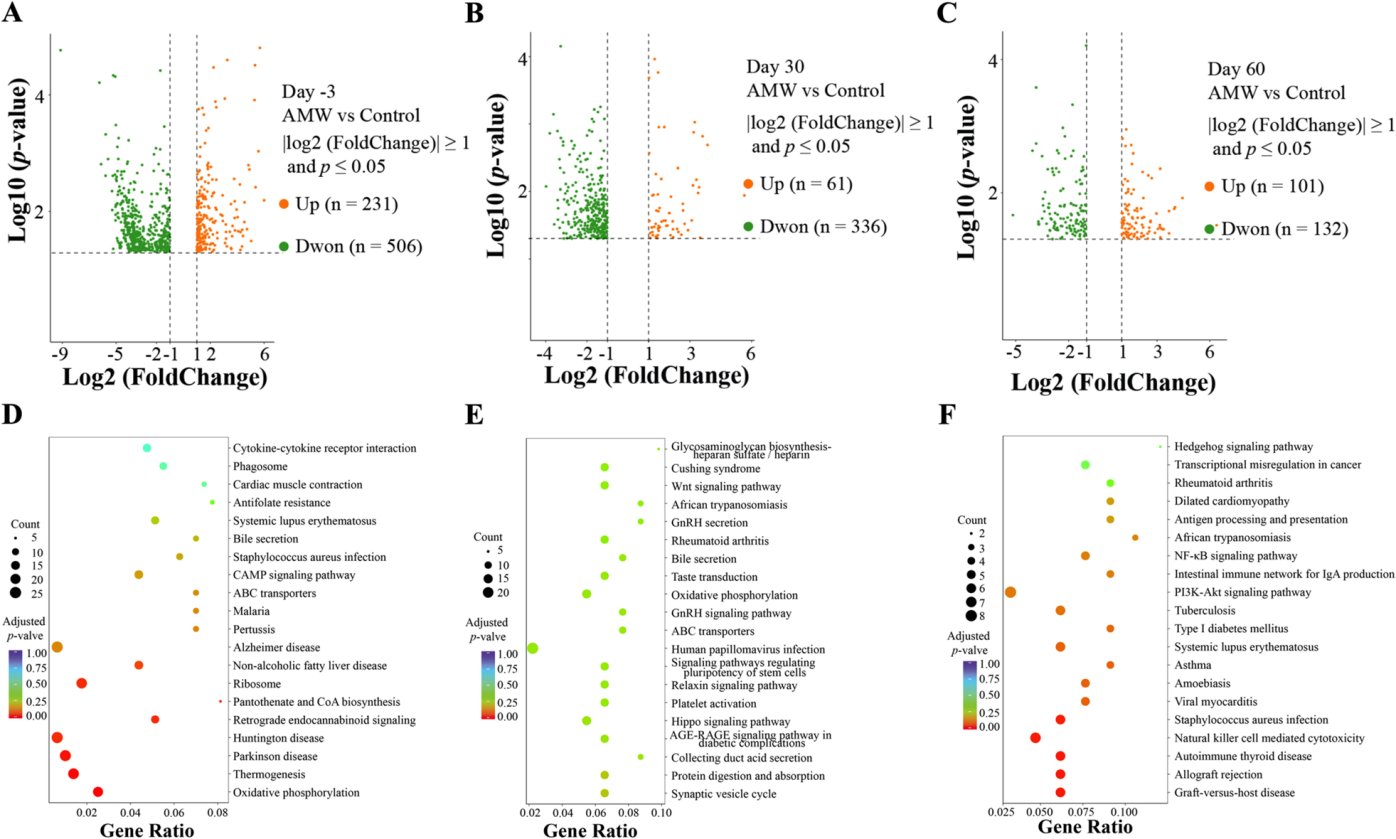
Visualization Results of Blood Transcriptomic Sequencing and Analysis. **A-C**, the volcano plots of the significantly up-regulated (orange) and down-regulated (green) genes between the AMW and Control groups on days -3 (**A**), 30 (**B**), and 60 (**C**). **D**-**F**, the bubble plots of the top 20 enriched KEGG pathways from the differentially expressed genes (DEGs) between the AMW and Control groups on days -3 (**A**), 30 (**B**), and 60 (**C**). In **D-F**, “Count” indicates the number of DEGs which were annotated to the corresponding pathway: a bigger value indicates more DEGs were enriched in this pathway; “Gene Ratio” indicates the ratio of the number of DEGs which was annotated to the corresponding pathways to the total number of DEGs: a bigger value indicates a higher enrichment level of DE Metas in this pathway; and “Adjusted *p*-values” indicates the significance corrected through multiple hypotheses. The color of symbols indicates their corresponding adjusted *p*-value: a bigger value indicates greater inspection reliability

**Fig. 4:**
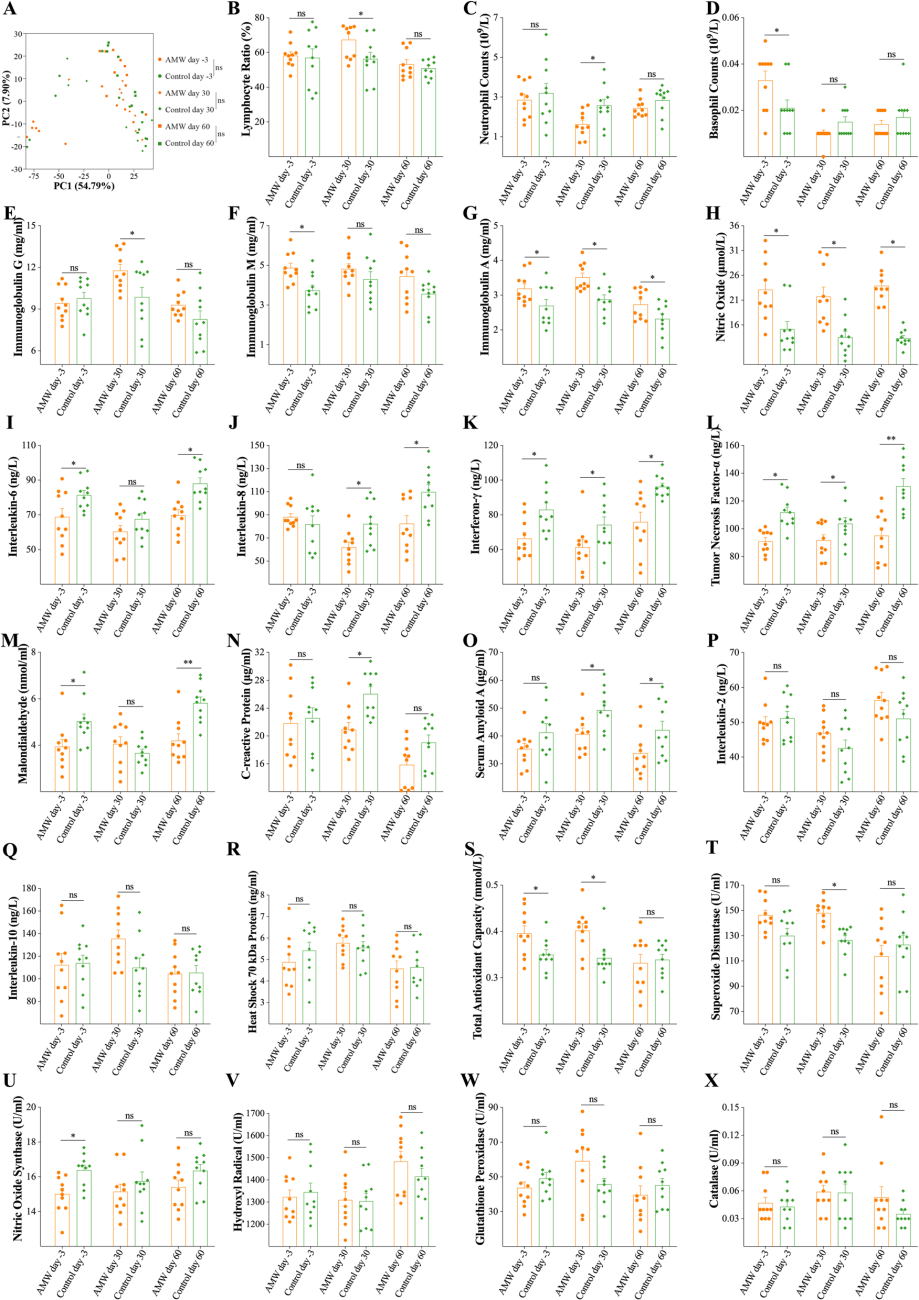
Comparison of the Serum Indicators Associated with the Obtained Enriched KEGG Pathways from the Transcriptomic Sequencing. **A**, results of the Principal Component Analysis (PCA) of gene expression data from calves’ blood based on fragments per kilobase million; **B**-**D**, ratio of lymphocyte counts to white cell counts (Lymphocyte Ratio), neutrophil counts, and basophil counts in the blood of calves in the AMW and Control groups at each time point; **E**-**R**, concentrations of immunoglobulin G, immunoglobulin M, immunoglobulin A, nitric oxide, interleukin (IL)-6, IL-8, interferon-γ, tumor necrosis factor-α, malondialdehyde, C-reactive protein, serum amyloid A, IL-2, IL-10, and heat shock 70 kDa protein in the blood of calves in the AMW and Control groups at each time point; **S-X**, activities of total antioxidant capacity, superoxide dismutase, nitric oxide synthase, hydroxyl radical, glutathione peroxidase, and catalase in the blood of calves in the AMW and Control groups at each time point. Data are expressed as the mean ± standard error mean (SEM). In **A**, the permutational multivariate analysis of variance was used to calculate the difference between the AMW and Control groups at each time point: ns, *p* > 0.05. In **B**, the Mann-Whitney U test was used to calculate the difference between the AMW and Control groups at each time point: ns, *p* > 0.05; *, *p* < 0.05. In **C**-**X**, the least significance difference method in the one-way analysis of variance was used to analyze the differences between the AMW and Control groups at each time point: ns, *p* > 0.05; *, *p* < 0.05; **, *p* < 0.01

The transcriptomic sequence of whole blood yielded a total of 416.2 G bp raw bases, with an average of 6.94 ± 0.44 G bases per sample. Following a thorough quality check, the samples produced 2,690,792,188 clean reads (150 bp), averaging 44,846,536.5 ± 3,022,240.28 reads per sample (Supplementary Table 5). Supplementary Table 6 provides detailed information on the DEGs detected in the blood samples of calves belonging to the AMW and Control groups on days -3, 30, and 60. There were 231, 61, and 101 genes in the blood of calves belonging to the AMW group that showed significantly higher (|log2 (FoldChange)| ≥ 1 and *p* ≤ 0.05) expression levels than those in the Control group on days -3, 30, and 60, respectively. In contrast, the expression levels of 506, 336, and132 genes were significantly lower (|log2 (FoldChange)| ≥ 1 and *p* ≤ 0.05) in the blood of calves in the AMW group compared to those in the Control group on days -3, 30, and 60, respectively (Fig. 3A-C). The PCA results showed that the gene expression patterns in the blood of calves from the AMW and Control groups at each time point could not be effectively separated (*p* > 0.05, Fig. 4A). Supplementary Table 7 shows detailed information on the DEGs enriched in KEGG pathways on days -3, 30, and 60. Fig. 3D-F shows the top 20 most enriched KEGG pathways between the AMW and Control groups on days -3, 30, and 60, respectively. On day -3, the DEGs between the AMW and Control groups were significantly enriched (adjusted *p* < 0.05) in 7 KEGG pathways: Oxidative Phosphorylation, Thermogenesis, Parkinson’s Disease, Huntington’s Disease, Retrograde Endocannabinoid Signaling, Pantothenate and CoA Biosynthesis, and Ribosome. On day 30, the DEGs between the AMW and Control groups were not significantly enriched (adjusted *p* > 0.05) in any KEGG pathway. On day 60, the DEGs between the AMW and Control groups were significantly enriched (adjusted *p* < 0.05) in 5 KEGG pathways: Graft-versus-host Disease, Allograft Rejection, Autoimmune Thyroid Disease, Natural Killer Cell-Mediated Cytotoxicity, and Staphylococcus Aureus Infection.

Table 2 presents the results of the RBT analysis. The blood samples of calves in the AMW group exhibited significant differences (*p* < 0.05) in the ratio of lymphocyte counts (LYM) to white blood cells (WBC; Fig. 4B), neutrophil counts (NEU; Fig. 4C), and basophil counts (BAS; Fig. 4D) compared to the Control group. The blood of calves in the AMW group exhibited higher levels of IgG on day 30, IgM on day -3, IgA on days -3, 30, and 60, and NO on days -3, 30, and 60 (*p* < 0.05; Fig. 4E-H), lower levels of IL-6 on days -3 and 60, IL-8 on days 30 and 60, IFN-γ on days -3, 30, and 60, TNF-α on days -3, 30, and 60, MDA on days -3 and 30, CRP on day 30, and SAA on days 30 and 60 (*p* < 0.05; Fig. 4I-O), and similar levels of IL-2, IL-10, and HSP70 (*p* > 0.05; Fig. 4P-R) on each day, compared to the Control group. Additionally, the calves in the AMW group demonstrated significantly elevated activities of T-AOC on days -3 and 30 and SOD on day 30 (*p* < 0.05; Fig. 4S and T), reduced activity of NOS (*p* < 0.05; Fig. 4U), and similar activities of OH, GSH-Px and CAT (*p* > 0.05; Fig. 4V-X) compared to the Control group.

**Table 2:** The results of the blood routine test analysis

**Items**	**day -3**		**day 30**		**day 60**	
	**AMW**	**Control**	**AMW**	**Control**	**AMW**	**Control**
**WBC** (10^9^/L)	8.08 ± 1.69	8.64 ± 1.23	7.96 ± 1.51	7.75 ± 0.92	6.94 ± 0.96	7.20 ± 1.60
**LYM** (10^9^/L)	4.73 ± 1.22	4.93 ± 1.48	5.45 ± 1.56	4.36 ± 0.98	3.75 ± 0.99	3.65 ± 0.90
**LYM Ratio** (%)	58.22 ± 6.95	56.98 ± 16.34	67.39 ± 10.19	56.32 ± 11.70	53.24 ± 8.27	50.84 ± 5.15
**MON** (10^9^/L)	0.40 ± 0.16	0.45 ± 0.19	0.67 ± 0.14	0.59 ± 0.11	0.50 ± 0.10	0.54 ± 0.15
**MON Ratio** (%)	4.98 ± 1.79	5.10 ± 2.23	8.60 ± 2.15	7.64 ± 1.49	7.23 ± 1.04	7.48 ± 1.29
**EOS** (10^9^/L)	0.06 ± 0.03	0.05 ± 0.05	0.21 ± 0.16	0.20 ± 0.15	0.23 ± 0.19	0.16 ± 0.11
**EOS Ratio** (%)	0.75 ± 0.38	0.55 ± 0.58	2.59 ± 2.03	2.54 ± 1.68	3.48 ± 3.16	2.19 ± 1.32
**NEU** (10^9^/L)	2.86 ± 0.86	3.20 ± 1.48	1.63 ± 0.63	2.59 ± 0.94	2.45 ± 0.41	2.84 ± 0.68
**NEU Ratio** (%)	35.63 ± 8.50	37.11 ± 17.47	21.29 ± 9.57	33.31 ± 11.74	35.85 ± 7.37	39.24 ± 4.20
**BAS** (10^9^/L)	0.03 ± 0.01	0.02 ± 0.01	0.01 ± 0.00	0.02 ± 0.01	0.01 ± 0.00	0.02 ± 0.01
**BAS Ratio** (%)	0.43 ± 0.20	0.26 ± 0.15	0.14 ± 0.10	0.19 ± 0.09	0.20 ± 0.07	0.24 ± 0.15
**RBC** (10^12^/L)	6.25 ± 0.53	6.07 ± 0.32	7.94 ± 1.61	8.51 ± 1.75	7.19 ± 0.61	7.28 ± 0.89
**HGB** (g/L)	122.20 ± 15.71	123.30 ± 16.54	96.20 ± 15.19	98.90 ± 16.84	94.60 ± 7.02	96.20 ± 8.68
**HCT** (%)	22.25 ± 2.35	20.91 ± 2.02	29.96 ± 4.80	30.08 ± 5.76	28.22 ± 2.97	29.17 ± 3.46
**MCV** (fL)	35.99 ± 1.78	35.75 ± 1.29	38.21 ± 4.06	35.53 ± 2.41	39.51 ± 4.91	40.30 ± 3.83
**MCH** (pg)	15.40 ± 2.24	13.35 ± 1.56	12.26 ± 1.00	11.73 ± 0.70	13.21 ± 0.92	13.03 ± 0.89
**MCHC** (g/L)	370.60 ± 27.61	331.10 ± 21.76	321.50 ± 10.93	330.20 ± 10.41	336.80 ± 23.04	344.40 ± 15.10

*WBC* White blood cell counts, *LYM* Lymphocyte counts, *LYM Ratio* The ratio of LYM to WBC, *MON* monocyte counts, *MON Ratio* The ratio of MON to WBC, *EOS* Eosinophil counts, *EOS Ratio* The ratio of EOS to WBC, *NEU* Neutrophil counts, *NUE Ratio* The ratio of NEU to WBC, *BAS* Basophil counts, *BAS Ratio* The ratio of BAS to WBC, *RBC* Red blood cell counts, *HGB* Hemoglobin concentration; *HCT* Hematocrit; and *PLT* Platelet counts; Results are expressed as the mean ± the standard deviation (SD)

### Profiles of serum small molecule metabolites

**Fig. 5:**
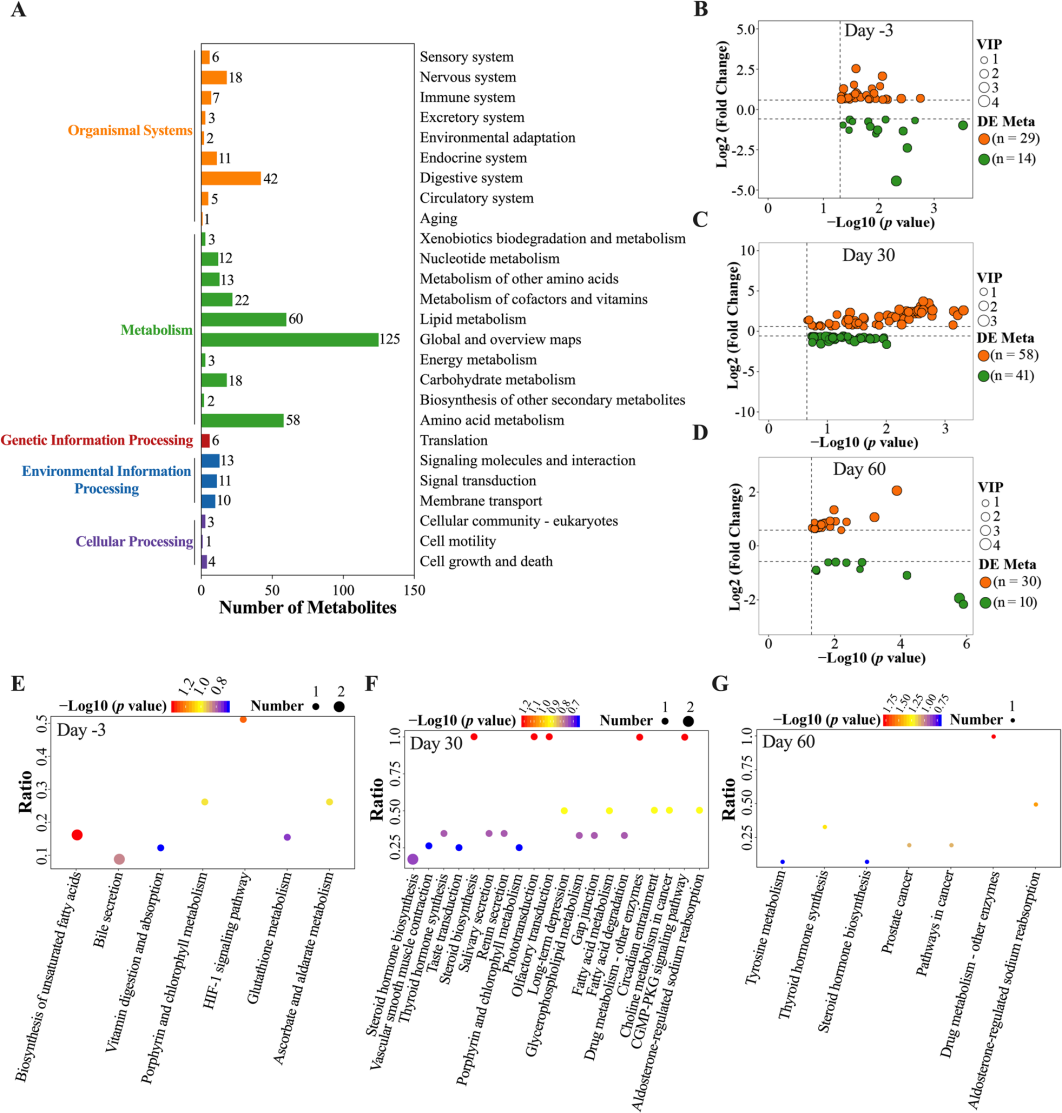
Visualization Results of Untargeted Metabolomics Sequencing and Analysis. **A**, annotation results of the detected metabolites in the KEGG database; **B**-**D**, volcano plots of the significantly up-regulated (orange) and down-regulated metabolites (green) between the AMW and Control groups on days -3 (**B**), 30 (**C**), and 60 (**D**), respectively; **E**-**G**, bubble plots of the top 20 enriched KEGG pathways from the differentially expressed metabolites between the AMW and Control groups on days -3 (**E**), 30 (**F**), and 60 (**G**), respectively. In **A**, the colored terms on the left represent the primary classification of these pathways with the same color, while the numbers on the right side of the column indicate the count of metabolites that belong to each signaling pathway. In E-G, “Number” indicates the number of DE Metas which were annotated to the corresponding pathway: a bigger value indicates more DE Metas enriched in this pathway; “Ratio” indicates the ratio of the number of DE Metas which were enriched in the corresponding pathway to the number of annotated metabolites in this pathway: a bigger value indicates a higher enrichment level of DE Metas in this pathway; and “*p*-values” indicates the significance calculated by hypergeometric inspection. The color of symbols indicates their corresponding *p*-value: a smaller value indicates greater inspection reliability. VIP, variable importance in the projection; DE Meta, differentially expressed metabolites

In the serum metabolomics analysis, 1092 metabolites were detected, with 459 classifieds into 26 KEGG pathways (Fig. 5A). In addition, a total of 211 of these 1092 detected metabolites were classified into 23 lipid maps (Supplementary Figure 3A). Between the AMW and Control groups, a total of 43, 99, and 40 DE Metas were identified on days -3, 30, and 60, respectively (Supplementary Table 8). Specifically, the AMW group exhibited significantly higher (VIP > 1, |log2(FoldChange)| ≥ 1, *p* < 0.05) relative concentrations of 29, 58, and 30 metabolites on days -3, 30, and 60, respectively, compared to the Control group. On the other hand, the AMW group displayed significantly lower (VIP > 1, |log2(FoldChange)| ≥ 1, *p* < 0.05) relative concentrations of 14, 41, and 10 metabolites on day -3, 30, and 60, respectively, as compared to the Control group (Fig. 5B-D). The results of the KEGG enrichment analysis of the DE Metas are shown in Supplementary Table 9 and Fig. 5E-G. The DE Metas exhibited a significant enrichment in one pathway, namely biosynthesis of unsaturated fatty acids, on day -3, while no significant enrichment was observed on day 30. However, on day 60, three pathways, namely drug metabolism-other enzymes, aldosterone-regulated sodium reabsorption, and thyroid hormone synthesis, showed significant enrichment between the AMW and Control groups.

**Fig. 6:**
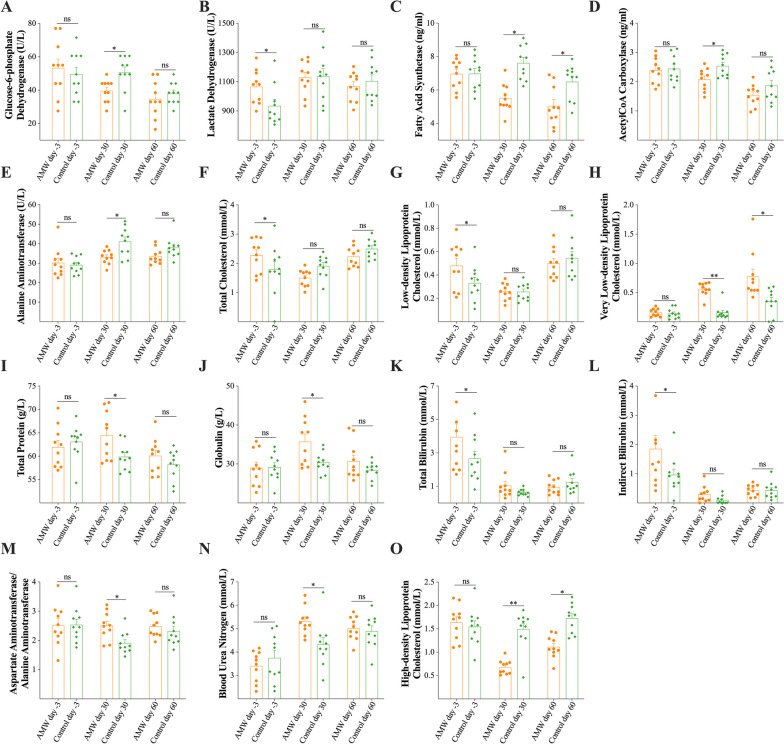
Comparison of the Indicators Associated with the Obtained Enriched KEGG Pathways from the Metabolomics Sequencing. **A-E**, the activities of glucose-6-phosphate dehydrogenase, lactate dehydrogenase, fatty acid synthetase, acetyl CoA carboxylase, alanine aminotransferase in the serum of calves in the AMW and Control groups at each time point; **F**-**O**, the concentrations of total cholesterol, low-density lipoprotein cholesterol, very low-density lipoprotein cholesterol, total protein, globulin, total bilirubin, indirect bilirubin, aspartate aminotransferase to alanine aminotransferase ratio, blood urea nitrogen, and high-density lipoprotein cholesterol in the serum of calves in the AMW and Control groups at each time point. Data are expressed as the mean ± the standard error mean (SEM). The least significance difference method in the one-way analysis of variance was used to analyze the differences between the AMW and Control groups at each time point: ns, *p* > 0.05; *, *p* < 0.05; **, *p* < 0.01

The serum of calves belonging to the AMW group demonstrated higher activities of G-6-PDH on day 30 and LDH on day -3 (*p* < 0.05; Fig. 6A and B). They also had lower activities of FAS on days 30 and 60, ACC on day 30, and ALT on day 30 (*p* < 0.05; Fig. 6C-E). However, the activities of PK, PFK, ALP, AST, GGT, and CK were similar on each day between the AMW group and the Control group (*p* > 0.05; Supplementary Figure 3B-G). Additionally, the AMW group exhibited significantly higher levels of TC on day -3, LDL-C on day -3, VLDL-C on days 30 and 60, TP on day 30, GLB on day 30, TBIL on day -3, IBIL on day -3, AST/ALT ratio on day 30, and BUN on day 30 (*p* < 0.05; Fig. 6F-N). In contrast, they had significantly lower levels of HDL-C on days 30 and 60 (*p* < 0.05; Fig. 6O). The serum levels of GLU, TG, NEFA, CRE, DBIL, ALB, ALB/GLB ratio, ACTH, COR, GH, and ADH were similar on each day between the AMW group and the Control group (*p* > 0.05; Supplementary Figure 3H-R).

### Relationships between the nasopharyngeal microbiome, blood transcriptome, and serum metabolome

**Fig. 7:**
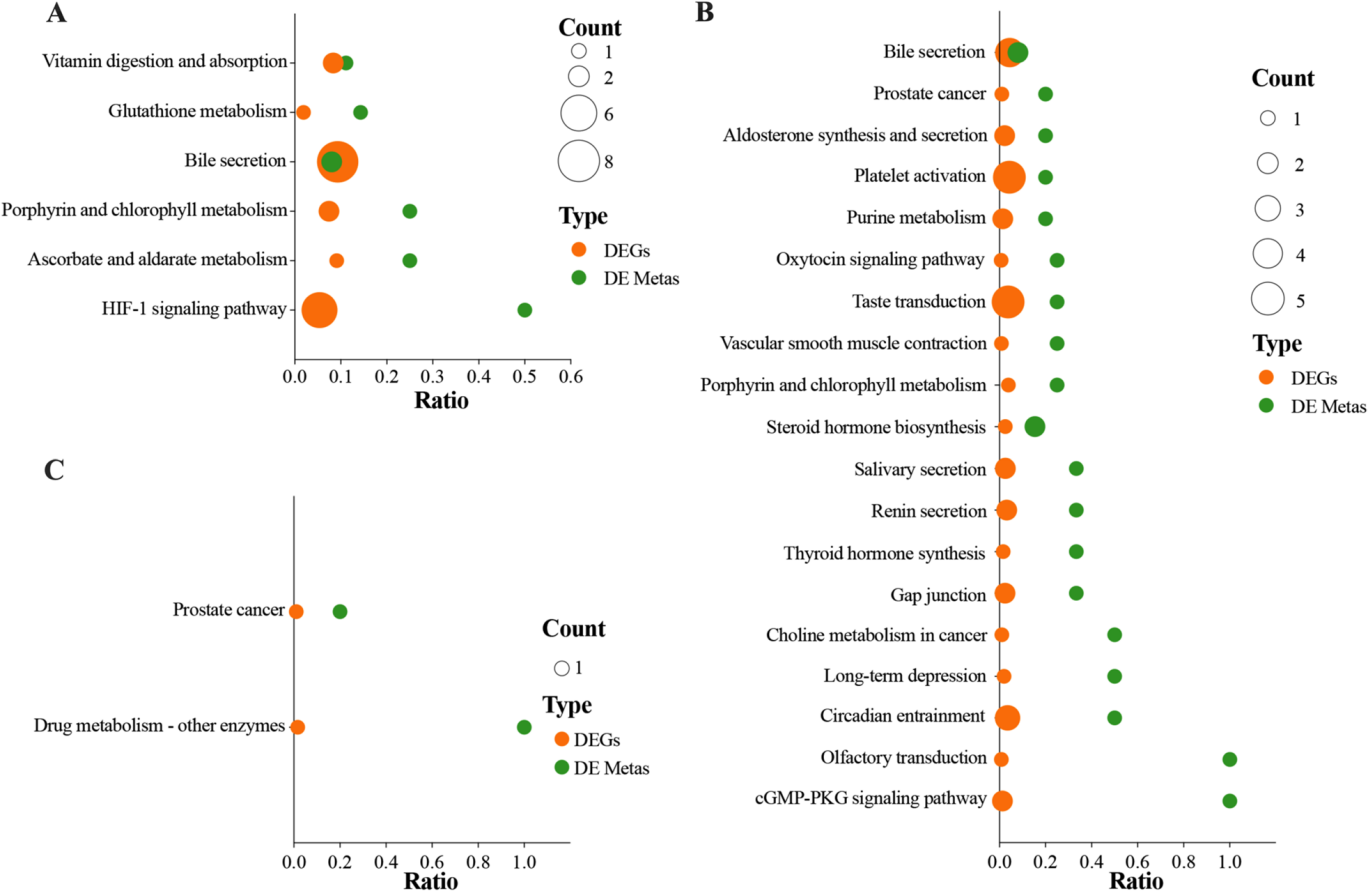
Enriched KEGG Pathways Shared by Differentially Expressed Genes and Differentially Expressed Metabolites in the Blood of Calves from the AMW and Control Groups. The color of bubbles (Type) represents the type of samples (orange for DEGs, green for DE Metas). The size of bubbles (Count) represents the numbers of the enriched DEGs or DE Metas: a bigger value indicates more DEGs or DE Metas that were enriched in this pathway. The Y-axis (Ratio) represents the ratio of counts of enriched DEGs or DE Metas to the total counts of genes or metabolites annotated in this pathway: a bigger value indicates a higher enrichment level of DEGs or de Metas in this pathway. DEGs, differentially expressed genes; DE Metas, differentially expressed metabolites

In the conjoint analysis of DEGs and DE Metas, the HIF-1 signaling pathway, ascorbate and aldarate metabolism, porphyrin and chlorophyll metabolism, bile secretion, glutathione metabolism, vitamin digestion and absorption were observed to be enriched on day -3 (Fig. 7A). On day 30, 19 pathways, namely cGMP-PKG signaling pathway, olfactory transduction, circadian entrainment, long-term depression, choline metabolism in cancer, gap junction, thyroid hormone synthesis, salivary secretion, steroid hormone biosynthesis, porphyrin and chlorophyll metabolism, vascular smooth muscle contraction, taste transduction, oxytocin signaling pathway, purine metabolism, platelet activation, aldosterone synthesis and secretion, prostate cancer, and bile secretion, were found to be enriched (Fig. 7B). However, only two pathways (drug metabolism-other enzymes and prostate cancer) were enriched on day 60 (Fig. 7C).

**Fig. 8:**
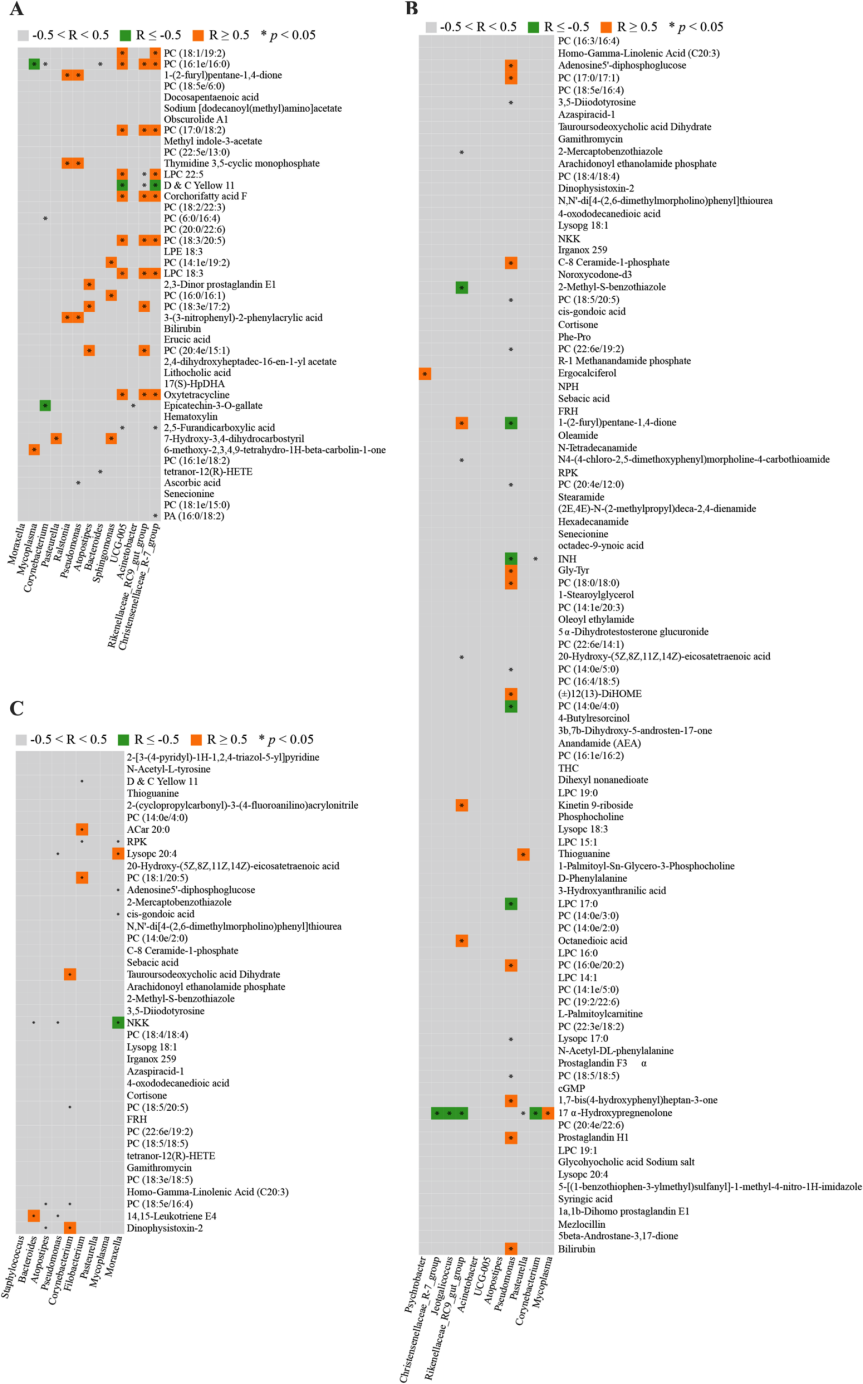
The Correlations between the Differentially Expressed Metabolites and the Nasopharyngeal Microorganisms with a Relative Abundance of Over 1% at the Genus Level. The Pearson’s Rank Correlation Analysis method was used to analyze the correlations between the differentially expressed metabolites and microorganisms with a relative abundance of over 1%. Squares with * indicate that there are significant correlations between their corresponding metabolites and microorganisms (*p* < 0.05). Orange squares represent that the correlation coefficients (R) between their corresponding metabolites and microorganisms ≥ 0.5, grey squares represent the correlation coefficients (R) between their corresponding metabolites and microorganism range from -0.5 to 0.5, and green squares represent that the correlation coefficients (R) between their corresponding metabolites and microorganisms ≤ 0.5. *, *p* < 0.05; R, correlation coefficients rho

The Spearman’s rank correlations between the DE Metas and the nasopharyngeal microbiota with a relative abundance of over 1% at the genus level were assessed. A total of 53 meaningful correlations (*p* < 0.05) were detected on day -3, of which 38 were positively correlated (R ≥ 0.5) and four were negatively correlated (R ≤ -0.5) (Fig. 8A). On day 30, 37 significant correlations (*p* < 0.05) were identified, of which 16 were positively correlated (R ≥ 0.5), and nine were negatively correlated (R ≤ -0.5) (Fig. 8B). At last, 20 remarkable correlations (*p* < 0.05) were found on day 60, one of which was negatively correlated (R ≤ -0.5) and six of which were positively correlated (R ≥ 0.5) (Fig. 8C).

## Discussion

Transporting young calves poses a significant reputational risk for the dairy and beef industry. These calves are often heifer calves destined for raising facilities to join dairy herds. Alternatively, they may be non-replacement calves transported either to the abattoir or to fattening farms for beef production. Cattle of any age experience stress before and after transportation, with young calves more prone to welfare compromises. This vulnerability is caused by several reasons, such as the fasting required for transportation, which increases the danger of energy depletion, hunger, hypoglycemia, and low glucose levels in the blood, particularly in young calves with little body fat reserves [1]. Transportation-induced BRD and growth suppression are the two main challenges that impede the progress of the cattle industry. While there are some descriptive reports regarding the advantageous outcomes of using additive agents on transported calves, there is a shortage of research that delves into the effects of additive agents on both BRD and calf growth performance, as well as the underlying mechanisms. This may be attributed to the significant workload, intricate clinical and laboratory outcomes, and the challenge of handling already stressed calves. Our study simultaneously evaluated the effects of AMW supplementation on the respiratory health status and growth performance of calves that had undergone 30 hours of road transportation, and we further explored its underlying mechanisms using a multi-omics approach.

Clinical scoring systems provide a reliable method for assessing the respiratory health of cattle, allowing farm managers to make proper treatment decisions without the need for expensive equipment or diagnostics, such as thoracic ultrasound and radiography. In order to ensure consistency and eliminate subjective differences, we assigned the responsibility of implementing a standardized scoring system to a single trained veterinarian for the entire 60-day period following the arrival of the cattle, referencing a previous study [22]. And our result demonstrated that calves in the AMW group exhibited notably lower scores in terms of respiration, appetite, ocular and ear, and nasal discharge (Fig. 1E-H) compared to those in the Control group during the 60 days after their arrival, indicating a better respiratory health status. The measurement of rectal temperature serves as an uncomplicated and practical gauge of the health status of cattle [28]. Although this difference was just 0.1 ℃, it indicated the significant difference in the rectal temperatures between calves in the AMW and Control group considering the large amount of data (30 calves per group, twice daily for 60 days). In our previous study, severe stimuli like dehorning surgery only increased the rectal temperature of calves by 0.4 ℃ [21]. Therefore, our findings demonstrated that calves categorized under the AMW group showed a decreased rectal temperature compared to those in the Control group (Fig. 1C). The findings mentioned above indicated that the AMW group calves had a higher level of respiratory tract health. Considering that the cattle are sensitive to the alteration of THI [29], we also monitored the THIs in the pens (Fig. 1B) throughout the 60 days. As expected, the rectal temperature and all five daily scores significantly correlated with the THIs (Supplementary Figure 1A-F), highlighting that the THI is a nonnegligible factor in investigations about cattle.

Our earlier research suggested that the nasopharyngeal microflora is more significantly impacted by the management procedures implemented after arrival rather than the transportation process [8]. Thus, to minimize transport stress, we opted to obtain nasopharyngeal swabs before transportation and on days 30 and 60 instead of upon arrival. Despite differences in the α diversity indexes of nasopharyngeal microbial communities between the AMW and Control groups of calves on days -3 and 60, the PCoA analysis revealed that the composition of their microbial communities was similar on both days (Fig. 2A-C). The relative abundance of Bacteroidota and Proteobacteria, which are two of the most abundant symbiota in the nasopharynx of calves [30, 31], was significantly higher in the calves from the AMW group compared to those in the Control group on days -3 and 30, respectively. Specifically, the calves supplied with AMW showed higher relative abundances of *UCG-0058*, *Bacteroides*, *Rikenellaceae_RC9_group*, and *Christensenellaceae_R-7_group* compared to those in the Control group on day -3, and a higher relative abundance of *Pseudomonas* compared to those who did not receive AMW on day 30 (Fig. 2E and H). These observations suggest that the nasopharyngeal microbial communities of calves were less affected by the commingling process before transportation, and were also less affected by the transportation process and post-transport management within 30 days after their arrival. Additionally, it is worth noting that the prevalence of *Histophilus somni*, a pathogen associated with BRD [32, 33], was considerably greater in the nasopharyngeal microflora of calves in the Control group compared to those in the AMW group on day -3 (Supplementary Figure 2H). Although the Chao1 index of the nasopharyngeal microbiota in the Control group was significantly greater than that in the AMW group on day 60, the microbiota composition was comparable between the two groups (Fig. 2J and K), indicating that the nasopharyngeal microbial communities of these calves tended to be stable, which was similar between the AMW group and Control group. This may explain the increased rectal temperature and daily scores (Fig. 1C-H) observed in the calves of the Control group within the previous month after their arrival. Overall, these findings demonstrate that the administration of AMW had a regulatory impact on the nasopharyngeal microorganisms of the transported calves.

After confirming the beneficial properties of AMW in enhancing respiratory health status, we were then curious about the underlying mechanisms that dictated its regulatory effects on the microbial population in the nasopharynx. Electrolytes are vital for cattle and have been used to reduce their transport stress by modifying their metabolic processes, altering the WBC levels, and increasing the live and carcass weights of transported calves [34]. In our investigation, we conducted whole blood transcriptomic analysis and observed that the WBCs of calves in the AMW group exhibited varying levels of activation in multiple pathways. These pathways include, but are not limited to, the cytokine-cytokine receptor interaction pathway, oxidative phosphorylation pathway, antigen processing and presentation pathway, NF-κB signaling pathway, the intestinal immune network for IgA production, and natural killer cell-mediated cytotoxicity pathway, compared to the Control group (Fig. 3D-F). The different levels of the LYM ratio, NEU count, and BAS count (Fig. 4B-D), as well as the levels of immunoglobulins and pro- and anti-inflammatory indicators (Fig. 4) in the blood of calves belonging to the AMW and Control groups also confirmed the results of transcriptomic sequencing. These outcomes suggest that supplying calves with AMW can enhance their peripheral immunity, paramount in preventing and managing BRD [35]. This may account for the observed differences in the respiratory health status and the nasopharyngeal microbiota composition of the calves.

The growth characteristics of calves that undergo transportation will be considerably impeded, leading to an escalation in both time and economic outlays. Feeder steers have been found to experience growth suppression up to 28 days after transportation [36]. In our studies, it was observed that the calves belonging to the AMW group exhibited a greater average daily gain of 0.498 kg/day compared to those in the Control group, who only showed an average daily gain of 0.122 kg/d (Fig. 1I). Additionally, it is essential to mention that the Control group's eight calves experienced a decrease in body weight, which suggests that their growth performance was significantly suppressed. The findings of serum untargeted metabolic sequencing indicate that the AMW supplementation resulted in the activation of several pathways, including bile secretion, biosynthesis of unsaturated fatty acids, and vitamin digestion and absorption (Fig. 5E-G), corroborated by the outcomes of serologic testing (Fig. 6). These pathways have been found to play pivotal roles in the absorption of fatty acids and the process of lipogenesis [37, 38], suggesting that AMW supplementation has resulted in a notable improvement in intestinal absorption and lipid synthesis abilities, which have contributed to the observed increase in growth performance.

In addition, 6 and 19 pathways were shared in the DEGs and DE Metas enriched KEGG pathways on days -3 and 30, respectively. Bile secretion was identified as a shared pathway on days -3 and 30 (Fig. 7A and B). However, only two pathways were shared on day 60 (Fig. 7C), indicating that the effects of AMW on the gene expression levels of WBC and metabolic processes were highest on day 30 and vanished on day 60, as the supplementation of AMW was cut off on day 30. Correspondingly, the correlation between the DE Metas and the nasopharyngeal microbiota on day -3 was stronger than on days 30 (Fig. 8). These results indicate that the AMW supplementation-induced DE Metas had more potent effects on the nasopharyngeal microbiota on day -3 than on day 30. The correlation between the DE Metas and nasopharyngeal microbiota was weak on day 60, highlighting the importance of continuous AMW supplementation.

## Conclusions

In summary, supplementation with AMW before and after transportation significantly lowered the rectal temperature and daily scores related to BRD in transported calves and increased their body weight gain. The results of the whole blood transcriptome, untargeted serum metabolism, RBT, and serologic testing collectively indicate that the calves provided with AMW demonstrated heightened peripheral immunity and intestinal absorption and lipogenesis capacities. The 16S rRNA gene microbiome revealed that calves supplied with AMW showed a different nasopharyngeal microbial community composition and structure, probably caused by enhanced peripheral immunity and altered metabolites. The findings further suggest that the beneficial outcomes of AMW are contingent upon its uninterrupted provision. The present research has successfully proposed an efficient strategy to mitigate the detrimental impacts of transportation-induced stress and has also shed light on its underlying mechanisms, which could contribute to the development of the cattle industry.

### Supplementary Information

**Additional file 1:** **Supplementary Figure 1.** Results of Correlation Analysis. A-F, the results of Pearson’s correlation analysis between the temperature and humidity indexes (THIs) and the rectal temperatures (A) and daily scores (E-F). The values of R^2^ and *p* were calculates using linear regression analysis in the SPSS software.

**Additional file 2:** **Supplementary Figure 2.** Visualization Results of 16S rRNA Gene Sequencing. A, the rarefaction curves of nasopharyngeal microbiota samples from calves; B, the number of observed species in the nasopharyngeal microbiota samples of calves; C and D, the relative abundance of the most ten abundant genera (C) and species (D), respectively, in the nasopharyngeal microbiota; E-H, the relative abundance of *Pasteurella multocida*, *Mannheimia haemolytica*, *Mycoplasmopsis bovis*, and *Histophilus somni* in the nasopharyngeal microbiota of calves; and I, the most abundant ten functions predicted using PICRUSt method of the microorganisms present in the nasopharynx of the calves. The data are expressed as the mean ± the standard error mean (SEM). In B, the least significance difference method in the one-way analysis of variance analysis was used to analyze the differences between the groups: ns, *p*> 0.05; *, *p* < 0.05. In E-H, the Mann-Whitney U test was used to analyze the difference in microbiota between the two groups at a specific time point: ns, non-significant; *, *p* < 0.05.

**Additional file 3:** **Supplementary Figure 3.** LipidMap Annotation Results of Metabolites and Comparison of the Indicators Associated with the Obtained Enriched KEGG Pathways from the Metabolomics Sequencing. A, annotation results of the detected metabolites in the LipidMap database. The colored terms on the left represent the primary classification of these pathways with same color, while the numbers on the right side of the column indicate the count of metabolites that belong to each signaling pathway. B-G, the activities of pyruvate kinase, phosphofructose kinase, alkaline phosphatase, aspartate aminotransferase, gamma-glutamyltransferase, and creatinine kinase in the serum of calves in the AMW and Control groups at each time point; H-R, the levels of glucose, triglyceride, non-esterified fatty acids, creatinine, direct bilirubin, albumin, albumin to globulin ratio, adrenocorticotropic hormone, cortisol, growth hormone, and antidiuretic hormone in the serum of calves in the AMW and Control groups at each time point. The data are expressed as the mean ± the standard error mean (SEM). In B-R, the Mann-Whitney U test (N) and least significance difference method in the one-way analysis of variance analysis (except for N) were used to analyze the differences between the groups at different time point: ns, *p* > 0.05.

**Additional file 4:** **Supplementary Table 1.** Composition and Nutrient Levels of Total Mixed Rations.

**Additional file 5:** **Supplementary Table 2.** Summary of 16S rRNA Gene Sequence Data Generated from Nasopharyngeal Swab Samples of the 20 Marked Calves at Three Time Points.

**Additional file 6:** **Supplementary Table 3.** Detailed Information on the Differences in Nasopharyngeal Microbiota of Calves between the AMW and Control Groups at Each Time Point (Excel Table).

**Additional file 7:** **Supplementary Table 4.** Detailed Information on the Contributions at the Genus Level to the Differences in the Nasopharyngeal Microbiota Community of Calves between the AMW and Control Groups at Each Time Point (Excel Table).

**Additional file 8:** **Supplementary Table 5.** Summary of Transcriptome Sequence Data Generated from the Whole Blood Samples of the 20 Marked Calves at Three Time Points.

**Additional file 9:** **Supplementary Table 6.** Detailed Information on the Differentially Expressed Genes between the AMW and Control Groups at Each Time Point (Excel Table).

**Additional file 10:** **Supplementary Table 7.** Results of KEGG Enrichment Analysis on Differentially Expressed Genes between the AMW and Control Groups at Each Time Point (Excel Table).

**Additional file 11:** **Supplementary Table 8.** Detailed Information on the Differentially Expressed Metabolites between the AMW and Control Groups at Each Time Point (Excel Table).

**Additional file 12:** **Supplementary Table 9.** Results of KEGG Enrichment Analysis on Differentially Expressed Metabolites between the AMW and Control Groups at Each Time Point (Excel Table).

## Data Availability

The raw data of the 16S rRNA gene microbiome sequence has been uploaded to the SRA database (BioProject: PRJNA992661; https://dataview.ncbi.nlm.nih.gov/object/PRJNA992661?reviewer=jp72seap3m5i9phrmjingik1o8); the raw data of the Whole-Blood transcriptome sequence has been uploaded to the SRA database (BioProject: PRJNA994857; https://dataview.ncbi.nlm.nih.gov/object/PRJNA994857?reviewer=l352dfudnt3m2oed8tt0h8b7i8); and the raw data of the untargeted metabolomics sequence has been uploaded to the Metabolights database (MTBLS8204; https://www.ebi.ac.uk/metabolights/reviewercb184a18-96b4-41c6-a1a5-26ff10335f8e).
