# Supplementary figures and images for: Multi-omics reveals that alkaline mineral water improves the respiratory health and growth performance of transported calves
Source: Microbiome. 2024 Mar 8;12:48. doi: 10.1186/s40168-023-01742-4 (PMC10921756; doi:10.1186/s40168-023-01742-4)

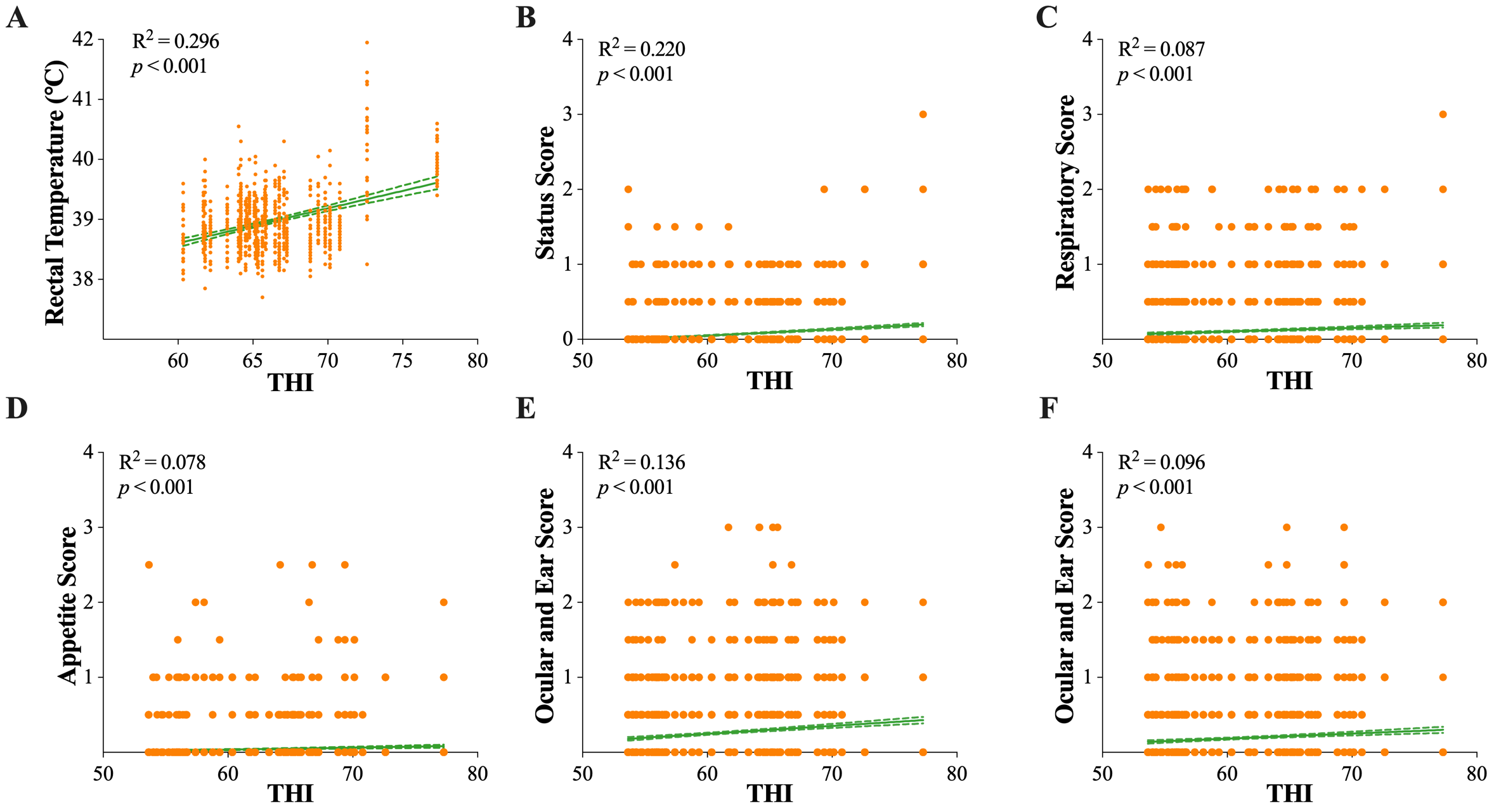

Supplement: Supplementary file 2 — Additional file 1: Supplementary Figure 1. Results of Correlation Analysis. A-F, the results of Pearson’s correlation analysis between the temperature and humidity indexes (THIs) and the rectal temperatures (A) and daily scores (E-F). The values of R2 and p were calculates using linear regression analysis in the SPSS software. [file 40168_2023_1742_MOESM1_ESM.tiff]

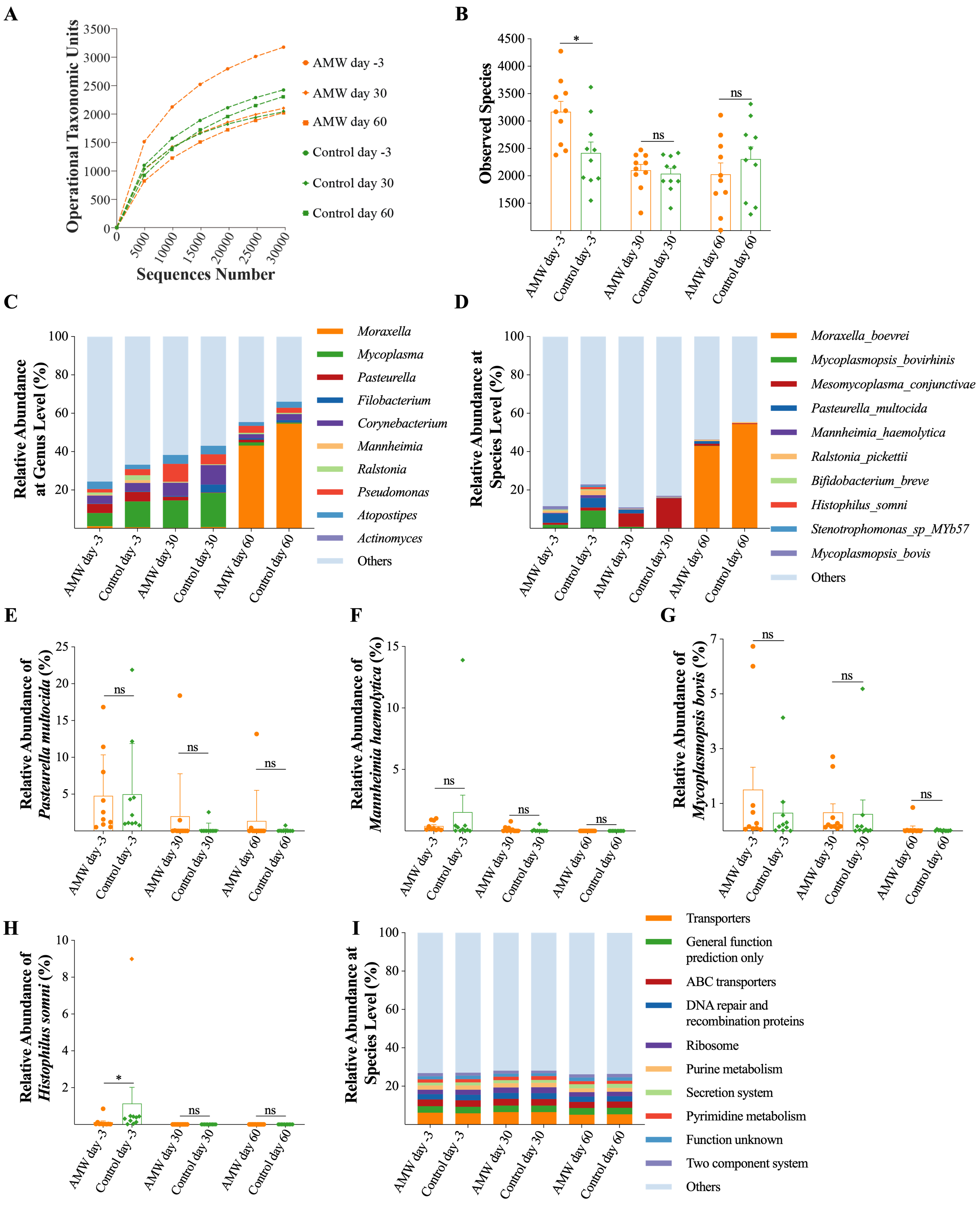

Supplement: Supplementary file 3 — Additional file 2: Supplementary Figure 2. Visualization Results of 16S rRNA Gene Sequencing. A, the rarefaction curves of nasopharyngeal microbiota samples from calves; B, the number of observed species in the nasopharyngeal microbiota samples of calves; C and D, the relative abundance of the most ten abundant genera (C) and species (D), respectively, in the nasopharyngeal microbiota; E-H, the relative abundance of Pasteurella multocida, Mannheimia haemolytica, Mycoplasmopsis bovis, and Histophilus somni in the nasopharyngeal microbiota of calves; and I, the most abundant ten functions predicted using PICRUSt method of the microorganisms present in the nasopharynx of the calves. The data are expressed as the mean ± the standard error mean (SEM). In B, the least significance difference method in the one-way analysis of variance analysis was used to analyze the differences between the groups: ns, p> 0.05; *, p < 0.05. In E-H, the Mann-Whitney U test was used to analyze the difference in microbiota between the two groups at a specific time point: ns, non-significant; *, p < 0.05. [file 40168_2023_1742_MOESM2_ESM.tiff]

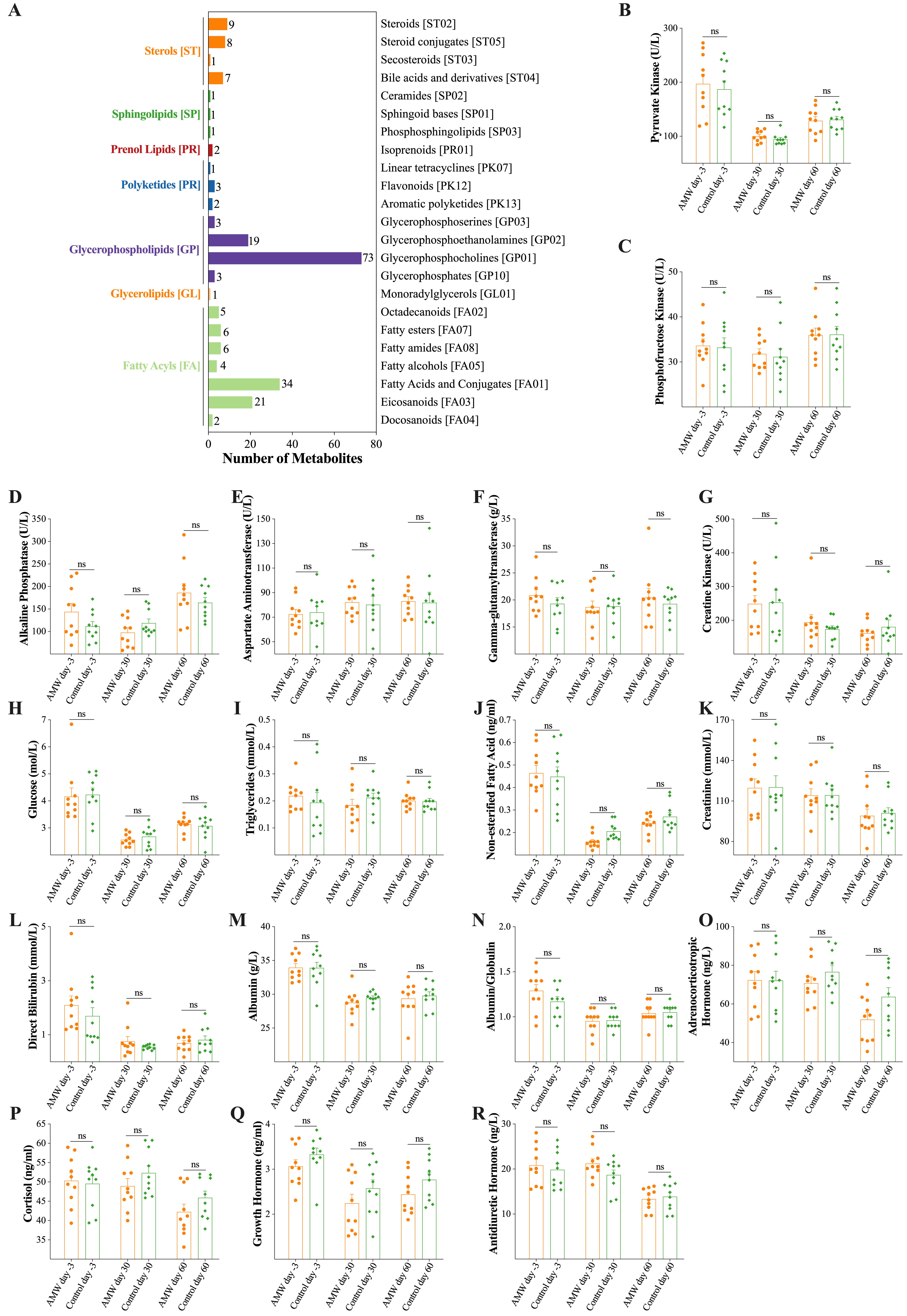

Supplement: Supplementary file 4 — Additional file 3: Supplementary Figure 3. LipidMap Annotation Results of Metabolites and Comparison of the Indicators Associated with the Obtained Enriched KEGG Pathways from the Metabolomics Sequencing. A, annotation results of the detected metabolites in the LipidMap database. The colored terms on the left represent the primary classification of these pathways with same color, while the numbers on the right side of the column indicate the count of metabolites that belong to each signaling pathway. B-G, the activities of pyruvate kinase, phosphofructose kinase, alkaline phosphatase, aspartate aminotransferase, gamma-glutamyltransferase, and creatinine kinase in the serum of calves in the AMW and Control groups at each time point; H-R, the levels of glucose, triglyceride, non-esterified fatty acids, creatinine, direct bilirubin, albumin, albumin to globulin ratio, adrenocorticotropic hormone, cortisol, growth hormone, and antidiuretic hormone in the serum of calves in the AMW and Control groups at each time point. The data are expressed as the mean ± the standard error mean (SEM). In B-R, the Mann-Whitney U test (N) and least significance difference method in the one-way analysis of variance analysis (except for N) were used to analyze the differences between the groups at different time point: ns, p > 0.05. [file 40168_2023_1742_MOESM3_ESM.tiff]
