# Supplementary material for: Multi-omics reveals that alkaline mineral water improves the respiratory health and growth performance of transported calves
Source: Microbiome. 2024 Mar 8;12:48. doi: 10.1186/s40168-023-01742-4 (PMC10921756; doi:10.1186/s40168-023-01742-4)
Supplement: Supplementary file 5 — Additional file 4: Supplementary Table 1. Composition and Nutrient Levels of Total Mixed Rations. [file 40168_2023_1742_MOESM4_ESM.docx]

Supplementary Table 1: **Composition and Nutrient Levels of Total Mixed Rations**

| **Diet Composition** | **Content** |
| --- | --- |
| Forage | 17 (%) |
| Silage | 55 (%) |
| Hay | 28 (%) |
| Total | 100 (%) |
|  |  |
| **Nutrient Levels** | **Content** |
| Metabolic Energy | 6.02 (MJ/kg) |
| Moisture Content | 13 (%) |
| Crude Protein (CP) | 20 (%) |
| Crude Fibre (CF) | 12 (%) |
| Crude Ash (CA) | 10 (%) |
| Sodium Chloride (NaCl) | 1 (%) |
| Methionine (Met) | 0.2 (%) |
| Calcium (Ca) | 1.5 (%) |
| Phosphorus (P) | 0.5 (%) |
