# Supplementary material for: Multi-omics reveals that alkaline mineral water improves the respiratory health and growth performance of transported calves
Source: Microbiome. 2024 Mar 8;12:48. doi: 10.1186/s40168-023-01742-4 (PMC10921756; doi:10.1186/s40168-023-01742-4)
Supplement: Supplementary file 6 — Additional file 5: Supplementary Table 2. Summary of 16S rRNA Gene Sequence Data Generated from Nasopharyngeal Swab Samples of the 20 Marked Calves at Three Time Points. [file 40168_2023_1742_MOESM5_ESM.docx]

Supplementary Table 2: **Summary of 16S rRNA Gene Sequence Data Generated from Nasopharyngeal Swab Samples of the 20 Marked Calves at Three Time Points**

| Sample Name | Raw Reads (#) | Raw Tags (#) | Clean Tags (#) | Effective Tags (#) | Base (nt) | AvgLen (nt) | Q20 (%) | Q30 (%) | GC Content (%) | Effective (%) |
| --- | --- | --- | --- | --- | --- | --- | --- | --- | --- | --- |
| Day (-3)-AMW-1 | 83,791 | 82,658 | 82,059 | 70,122 | 17,890,372 | 255 | 99.09 | 97.31 | 53.33 | 83.69 |
| Day (-3)-AMW-2 | 79,526 | 77,592 | 77,081 | 71,575 | 17,907,536 | 250 | 99.05 | 97.11 | 53.66 | 90 |
| Day (-3)-AMW-3 | 86,976 | 84,791 | 84,224 | 78,048 | 19,498,984 | 250 | 98.99 | 96.97 | 53.12 | 89.74 |
| Day (-3)-AMW-4 | 85,684 | 84,115 | 83,451 | 78,620 | 19,825,524 | 252 | 98.93 | 96.85 | 52.72 | 91.76 |
| Day (-3)-AMW-5 | 85,915 | 84,411 | 83,812 | 79,302 | 19,904,857 | 251 | 99.11 | 97.29 | 51.25 | 92.3 |
| Day (-3)-AMW-6 | 81,760 | 79,990 | 79,453 | 73,624 | 18,509,589 | 251 | 99.05 | 97.11 | 52.18 | 90.05 |
| Day (-3)-AMW-7 | 91,066 | 88,901 | 88,174 | 82,968 | 20,840,555 | 251 | 98.98 | 97.03 | 52.9 | 91.11 |
| Day (-3)-AMW-8 | 59,911 | 55,737 | 54,969 | 52,145 | 13,140,243 | 252 | 98.96 | 96.93 | 50.55 | 87.04 |
| Day (-3)-AMW-9 | 86,807 | 86,145 | 85,610 | 79,509 | 20,090,548 | 253 | 99.01 | 97.03 | 52.89 | 91.59 |
| Day (-3)-AMW-10 | 85,301 | 82,581 | 81,743 | 77,584 | 19,577,289 | 252 | 99.08 | 97.22 | 51.9 | 90.95 |
| Day (30)-AMW-1 | 88,870 | 88,048 | 87,391 | 77,845 | 19,694,504 | 253 | 99.31 | 97.98 | 51.95 | 87.59 |
| Day (30)-AMW-2 | 92,585 | 92,025 | 91,679 | 87,316 | 22,085,543 | 253 | 99.26 | 97.9 | 50.82 | 94.31 |
| Day (30)-AMW-3 | 82,487 | 79,865 | 79,469 | 74,919 | 18,808,898 | 251 | 99.23 | 97.79 | 51.9 | 90.83 |
| Day (30)-AMW-4 | 85,916 | 85,510 | 85,291 | 78,224 | 19,546,895 | 250 | 99.26 | 97.85 | 50.99 | 91.05 |
| Day (30)-AMW-5 | 90,762 | 89,885 | 89,518 | 82,135 | 20,725,137 | 252 | 99.2 | 97.72 | 52.23 | 90.49 |
| Day (30)-AMW-6 | 82,186 | 80,035 | 79,701 | 73,452 | 18,462,607 | 251 | 99.23 | 97.78 | 52.13 | 89.37 |
| Day (30)-AMW-7 | 85,295 | 84,303 | 84,046 | 78,055 | 19,429,094 | 249 | 99.18 | 97.63 | 51.81 | 91.51 |
| Day (30)-AMW-8 | 87,376 | 85,088 | 84,715 | 79,763 | 19,996,380 | 251 | 99.23 | 97.81 | 51.66 | 91.29 |
| Day (30)-AMW-9 | 89,076 | 81,080 | 80,710 | 75,696 | 18,767,352 | 248 | 99.16 | 97.71 | 51.51 | 84.98 |
| Day (30)-AMW-10 | 78,584 | 77,666 | 77,410 | 72,420 | 18,204,971 | 251 | 99.25 | 97.82 | 48.97 | 92.16 |
| Day (60)-AMW-1 | 90,739 | 89,160 | 88,577 | 82,992 | 20,904,173 | 252 | 99.36 | 97.96 | 51.67 | 91.46 |
| Day (60)-AMW-2 | 81,808 | 80,462 | 80,001 | 75,866 | 19,127,764 | 252 | 99.44 | 98.18 | 51.95 | 92.74 |
| Day (60)-AMW-3 | 88,262 | 86,020 | 85,567 | 81,393 | 20,479,764 | 252 | 99.44 | 98.17 | 52.6 | 92.22 |
| Day (60)-AMW-4 | 92,957 | 91,577 | 91,142 | 88,801 | 22,404,019 | 252 | 99.5 | 98.36 | 52.13 | 95.53 |
| Day (60)-AMW-5 | 88,476 | 88,074 | 87,702 | 85,024 | 21,456,301 | 252 | 99.53 | 98.43 | 51.99 | 96.1 |
| Day (60)-AMW-6 | 79,967 | 78,480 | 78,117 | 74,760 | 18,847,210 | 252 | 99.48 | 98.25 | 51.98 | 93.49 |
| Day (60)-AMW-7 | 83,124 | 81,973 | 81,664 | 78,137 | 19,523,403 | 250 | 99.38 | 98.02 | 52.06 | 94 |
| Day (60)-AMW-8 | 90,183 | 89,795 | 89,426 | 82,639 | 20,792,528 | 252 | 99.4 | 98.05 | 52.2 | 91.63 |
| Day (60)-AMW-9 | 92,507 | 90,347 | 89,831 | 85,281 | 21,540,050 | 253 | 99.47 | 98.26 | 51.9 | 92.19 |
| Day (60)-AMW-10 | 85,513 | 83,989 | 83,615 | 77,103 | 19,142,354 | 248 | 99.39 | 98.01 | 52.56 | 90.17 |
| Day (-3)-Control-1 | 90,666 | 89,818 | 89,293 | 85,476 | 21,550,891 | 252 | 99.1 | 97.26 | 51.92 | 94.28 |
| Day (-3)-Control-2 | 69,146 | 66,303 | 65,668 | 62,323 | 15,715,786 | 252 | 99.16 | 97.42 | 51.84 | 90.13 |
| Day (-3)-Control-3 | 87,985 | 87,008 | 86,472 | 83,274 | 20,933,725 | 251 | 98.93 | 96.91 | 52.71 | 94.65 |
| Day (-3)-Control-4 | 85,833 | 84,525 | 83,831 | 81,237 | 20,561,366 | 253 | 98.96 | 96.86 | 48.15 | 94.65 |
| Day (-3)-Control-5 | 75,966 | 72,321 | 71,398 | 68,480 | 17,266,843 | 252 | 98.99 | 97.04 | 52.66 | 90.15 |
| Day (-3)-Control-6 | 94,810 | 92,888 | 92,133 | 87,354 | 21,965,265 | 251 | 98.84 | 96.65 | 52.56 | 92.14 |
| Day (-3)-Control-7 | 86,312 | 85,547 | 85,180 | 71,231 | 18,099,494 | 254 | 99.21 | 97.62 | 52.91 | 82.53 |
| Day (-3)-Control-8 | 55,934 | 54,263 | 53,748 | 51,097 | 12,890,289 | 252 | 99.11 | 97.32 | 51.07 | 91.35 |
| Day (-3)-Control-9 | 86,706 | 86,111 | 85,642 | 80,984 | 20,262,399 | 250 | 98.94 | 96.89 | 52.97 | 93.4 |
| Day (-3)-Control-10 | 68,005 | 58,968 | 58,088 | 54,370 | 13,521,394 | 249 | 98.94 | 96.92 | 52.18 | 79.95 |
| Day (30)-Control-1 | 81,032 | 75,704 | 75,192 | 67,811 | 16,980,367 | 250 | 99.16 | 97.69 | 51.62 | 83.68 |
| Day (30)-Control-2 | 89,161 | 89,032 | 88,811 | 85,038 | 21,421,624 | 252 | 99.28 | 97.87 | 51.56 | 95.38 |
| Day (30)-Control-3 | 81,512 | 81,281 | 80,898 | 75,095 | 19,009,654 | 253 | 99.18 | 97.69 | 52.62 | 92.13 |
| Day (30)-Control-4 | 83,633 | 83,564 | 83,365 | 76,629 | 19,300,958 | 252 | 99.27 | 97.88 | 50.72 | 91.63 |
| Day (30)-Control-5 | 58,430 | 57,460 | 57,058 | 55,203 | 13,941,275 | 253 | 99.16 | 97.66 | 46.68 | 94.48 |
| Day (30)-Control-6 | 78,778 | 76,852 | 76,327 | 71,608 | 18,078,849 | 252 | 99.22 | 97.75 | 51.52 | 90.9 |
| Day (30)-Control-7 | 93,395 | 93,016 | 92,641 | 86,323 | 21,829,030 | 253 | 99.24 | 97.81 | 51.01 | 92.43 |
| Day (30)-Control-8 | 59,898 | 58,616 | 58,288 | 54,955 | 13,873,683 | 252 | 99.19 | 97.75 | 52.38 | 91.75 |
| Day (30)-Control-9 | 90,840 | 88,656 | 88,250 | 84,686 | 21,344,111 | 252 | 99.09 | 97.41 | 49.77 | 93.23 |
| Day (30)-Control-10 | 85,296 | 84,002 | 83,445 | 79,974 | 20,192,447 | 252 | 99.19 | 97.74 | 51.47 | 93.76 |
| Day (60)-Control-1 | 90,805 | 90,700 | 90,428 | 87,815 | 22,118,688 | 252 | 99.5 | 98.32 | 52.32 | 96.71 |
| Day (60)-Control-2 | 90,741 | 89,497 | 89,126 | 85,671 | 21,557,986 | 252 | 99.44 | 98.18 | 52.46 | 94.41 |
| Day (60)-Control-3 | 83,429 | 82,893 | 82,538 | 76,976 | 19,316,441 | 251 | 99.44 | 98.14 | 51.88 | 92.27 |
| Day (60)-Control-4 | 88,158 | 87,731 | 87,404 | 82,565 | 20,776,478 | 252 | 99.48 | 98.27 | 52.31 | 93.66 |
| Day (60)-Control-5 | 88,810 | 88,739 | 88,328 | 81,650 | 20,515,649 | 251 | 99.27 | 97.7 | 53.07 | 91.94 |
| Day (60)-Control-6 | 89,224 | 87,204 | 86,634 | 84,261 | 21,281,888 | 253 | 99.45 | 98.22 | 52.16 | 94.44 |
| Day (60)-Control-7 | 86,072 | 83,941 | 83,375 | 80,868 | 20,397,354 | 252 | 99.45 | 98.22 | 52.31 | 93.95 |
| Day (60)-Control-8 | 85,926 | 84,870 | 84,453 | 80,410 | 20,250,654 | 252 | 99.37 | 98 | 52.37 | 93.58 |
| Day (60)-Control-9 | 79,256 | 77,339 | 76,959 | 70,196 | 17,232,627 | 245 | 99.12 | 97.32 | 52.6 | 88.57 |
| Day (60)-Control-10 | 86,829 | 85,735 | 85,275 | 80,023 | 20,098,670 | 251 | 99.37 | 97.97 | 52.68 | 92.16 |
| Total | 5,035,998 | 4,934,887 | 4,906,396 | 4,608,901 | 1,159,410,329 | / | / | / | / | / |
| Mean | 83,933 | 82,248 | 81,773 | 76,815 | 19,323,505 | 251.48 | 99.22 | 97.65 | 51.87 | 91.43 |
| Standard Deviation | 8524.29 | 9125.30 | 9166.55 | 8887.45 | 2250364.83 | 1.53 | 0.18 | 0.47 | 1.16 | 3.23 |

Note: "Raw Tags" represents the tag sequence obtained by splicing; "Clean Tags" sequence is filtered from the "Raw Tags" to eliminate short length and low-quality data; "Effective Tags" sequence is the final output after removing chimeric data; "Base" number represents the number of bases in the final "Effective Tags"; "AvgLen" is the average length of "Effective Tags"; "Q20" and "Q30" indicate the percentage of base mass values greater than 20 (error rates less than 1%) and 30 (error rates less than 0.1%), respectively, in “Effective Tags”; "GC Content (%)" represents the content of GC bases in "Effective Tags"; "Effective (%)" represents the percentage of "Effective Tags" obtained compared to the number of "Raw Tags".
