# Supplementary material for: Multi-omics reveals that alkaline mineral water improves the respiratory health and growth performance of transported calves
Source: Microbiome. 2024 Mar 8;12:48. doi: 10.1186/s40168-023-01742-4 (PMC10921756; doi:10.1186/s40168-023-01742-4)
Supplement: Supplementary file 9 — Additional file 8: Supplementary Table 5. Summary of Transcriptome Sequence Data Generated from the Whole Blood Samples of the 20 Marked Calves at Three Time Points. [file 40168_2023_1742_MOESM8_ESM.docx]

Supplementary Table 5: **Summary of Transcriptome Sequence Data Generated from the Whole Blood Samples of the 20 Marked Calves at Three Time Points**

| Sample Name | Raw Reads (#) | Raw Bases (G bp) | Clean Reads (#) | Clean Bases (G bp) | Error Rate | Q20 (%) | Q30 (%) | GC Content (%) |
| --- | --- | --- | --- | --- | --- | --- | --- | --- |
| Day (-3)-AMW-1 | 47725976 | 7.16 | 46635006 | 7.00 | 0.03 | 97.46 | 93.33 | 54.10 |
| Day (-3)-AMW-2 | 47035366 | 7.06 | 45640086 | 6.85 | 0.03 | 97.42 | 93.16 | 59.39 |
| Day (-3)-AMW-3 | 45312130 | 6.80 | 43839164 | 6.58 | 0.03 | 97.73 | 93.99 | 55.57 |
| Day (-3)-AMW-4 | 42152942 | 6.32 | 41148676 | 6.17 | 0.03 | 97.59 | 93.66 | 53.36 |
| Day (-3)-AMW-5 | 46717126 | 7.01 | 45560266 | 6.83 | 0.03 | 97.79 | 94.08 | 58.54 |
| Day (-3)-AMW-6 | 53415974 | 8.01 | 52733262 | 7.91 | 0.02 | 98.19 | 94.87 | 59.28 |
| Day (-3)-AMW-7 | 55707780 | 8.36 | 54958914 | 8.24 | 0.02 | 98.08 | 94.62 | 59.04 |
| Day (-3)-AMW-8 | 46342984 | 6.95 | 45312432 | 6.80 | 0.03 | 97.53 | 93.38 | 54.78 |
| Day (-3)-AMW-9 | 55736750 | 8.36 | 55110672 | 8.27 | 0.02 | 98.21 | 94.90 | 57.88 |
| Day (-3)-AMW-10 | 48058588 | 7.21 | 47349678 | 7.10 | 0.02 | 98.27 | 95.02 | 58.11 |
| Day (30)-AMW-1 | 46850160 | 7.03 | 45996732 | 6.90 | 0.03 | 97.65 | 93.57 | 52.52 |
| Day (30)-AMW-2 | 45433376 | 6.82 | 44640054 | 6.70 | 0.03 | 97.61 | 93.51 | 52.52 |
| Day (30)-AMW-3 | 47645632 | 7.15 | 46073072 | 6.91 | 0.03 | 97.96 | 94.13 | 51.26 |
| Day (30)-AMW-4 | 47111656 | 7.07 | 46303382 | 6.95 | 0.03 | 97.70 | 93.78 | 53.32 |
| Day (30)-AMW-5 | 45727824 | 6.86 | 44450088 | 6.67 | 0.03 | 97.83 | 94.00 | 51.08 |
| Day (30)-AMW-6 | 44605830 | 6.69 | 43511450 | 6.53 | 0.03 | 97.65 | 93.60 | 51.98 |
| Day (30)-AMW-7 | 42082816 | 6.31 | 41196724 | 6.18 | 0.03 | 97.78 | 93.85 | 52.76 |
| Day (30)-AMW-8 | 47644472 | 7.15 | 46498244 | 6.97 | 0.02 | 97.98 | 94.31 | 55.80 |
| Day (30)-AMW-9 | 46133980 | 6.92 | 44730308 | 6.71 | 0.03 | 97.60 | 93.52 | 53.23 |
| Day (30)-AMW-10 | 45521922 | 6.83 | 44071304 | 6.61 | 0.03 | 97.60 | 93.53 | 52.26 |
| Day (60)-AMW-1 | 53607210 | 8.04 | 52061036 | 7.81 | 0.03 | 96.99 | 92.20 | 54.72 |
| Day (60)-AMW-2 | 46218582 | 6.93 | 44163604 | 6.62 | 0.03 | 97.35 | 93.09 | 54.12 |
| Day (60)-AMW-3 | 45756168 | 6.86 | 44585766 | 6.69 | 0.03 | 97.53 | 93.43 | 54.72 |
| Day (60)-AMW-4 | 45143724 | 6.77 | 43774864 | 6.57 | 0.03 | 97.34 | 92.96 | 52.46 |
| Day (60)-AMW-5 | 45267352 | 6.79 | 43394228 | 6.51 | 0.03 | 97.48 | 93.18 | 52.68 |
| Day (60)-AMW-6 | 46925156 | 7.04 | 44984308 | 6.75 | 0.03 | 97.40 | 92.99 | 52.63 |
| Day (60)-AMW-7 | 45318180 | 6.80 | 42236408 | 6.34 | 0.03 | 97.32 | 92.93 | 52.28 |
| Day (60)-AMW-8 | 45444748 | 6.82 | 41629118 | 6.24 | 0.02 | 98.00 | 94.30 | 50.37 |
| Day (60)-AMW-9 | 35555752 | 5.33 | 35485482 | 5.32 | 0.02 | 98.25 | 94.94 | 49.73 |
| Day (60)-AMW-10 | 47084890 | 7.06 | 46969780 | 7.05 | 0.03 | 97.50 | 93.40 | 52.59 |
| Day (-3)-Control-1 | 44364148 | 6.65 | 43374042 | 6.51 | 0.03 | 97.84 | 94.16 | 57.35 |
| Day (-3)-Control-2 | 44133418 | 6.62 | 43263430 | 6.49 | 0.03 | 97.66 | 93.78 | 54.82 |
| Day (-3)-Control-3 | 47359874 | 7.10 | 46122014 | 6.92 | 0.02 | 97.91 | 94.30 | 51.44 |
| Day (-3)-Control-4 | 42623960 | 6.39 | 41284748 | 6.19 | 0.02 | 97.89 | 94.33 | 59.20 |
| Day (-3)-Control-5 | 44631590 | 6.69 | 43543350 | 6.53 | 0.03 | 97.85 | 94.19 | 59.12 |
| Day (-3)-Control-6 | 44205424 | 6.63 | 42764322 | 6.41 | 0.02 | 97.93 | 94.35 | 57.65 |
| Day (-3)-Control-7 | 46744922 | 7.01 | 45797710 | 6.87 | 0.03 | 97.72 | 93.80 | 51.95 |
| Day (-3)-Control-8 | 45390362 | 6.81 | 44044036 | 6.61 | 0.03 | 97.62 | 93.60 | 51.64 |
| Day (-3)-Control-9 | 47681370 | 7.15 | 46485284 | 6.97 | 0.03 | 97.83 | 94.09 | 50.83 |
| Day (-3)-Control-10 | 45241002 | 6.79 | 43869740 | 6.58 | 0.03 | 97.69 | 93.72 | 51.24 |
| Day (30)-Control-1 | 45343166 | 6.80 | 44214746 | 6.63 | 0.03 | 97.81 | 94.03 | 55.12 |
| Day (30)-Control-2 | 47165726 | 7.07 | 45239012 | 6.79 | 0.03 | 97.71 | 93.58 | 47.71 |
| Day (30)-Control-3 | 45837654 | 6.88 | 44967474 | 6.75 | 0.03 | 97.13 | 92.55 | 51.53 |
| Day (30)-Control-4 | 45741464 | 6.86 | 44737546 | 6.71 | 0.03 | 97.74 | 93.85 | 55.76 |
| Day (30)-Control-5 | 45675134 | 6.85 | 43345732 | 6.5 | 0.03 | 97.47 | 93.34 | 51.42 |
| Day (30)-Control-6 | 45897454 | 6.88 | 44914628 | 6.74 | 0.03 | 97.55 | 93.40 | 52.78 |
| Day (30)-Control-7 | 46651232 | 7.00 | 45553354 | 6.83 | 0.03 | 97.60 | 93.53 | 52.85 |
| Day (30)-Control-8 | 45596510 | 6.84 | 44467742 | 6.67 | 0.03 | 97.61 | 93.46 | 50.80 |
| Day (30)-Control-9 | 45648378 | 6.85 | 44648254 | 6.70 | 0.03 | 97.95 | 94.22 | 55.16 |
| Day (30)-Control-10 | 47037400 | 7.06 | 45711956 | 6.86 | 0.03 | 97.60 | 93.52 | 51.64 |
| Day (60)-Control-1 | 47571106 | 7.14 | 44622930 | 6.69 | 0.03 | 97.01 | 92.35 | 53.23 |
| Day (60)-Control-2 | 46204392 | 6.93 | 44023854 | 6.60 | 0.03 | 97.15 | 92.63 | 53.69 |
| Day (60)-Control-3 | 46768744 | 7.02 | 45175524 | 6.78 | 0.03 | 97.54 | 93.42 | 53.90 |
| Day (60)-Control-4 | 45845422 | 6.88 | 44247048 | 6.64 | 0.03 | 97.15 | 92.63 | 54.29 |
| Day (60)-Control-5 | 45425664 | 6.81 | 44159208 | 6.62 | 0.03 | 97.39 | 93.13 | 53.25 |
| Day (60)-Control-6 | 45298576 | 6.79 | 43151424 | 6.47 | 0.03 | 97.39 | 93.08 | 51.06 |
| Day (60)-Control-7 | 47525286 | 7.13 | 45919292 | 6.89 | 0.03 | 97.33 | 92.82 | 53.63 |
| Day (60)-Control-8 | 46297072 | 6.94 | 42140220 | 6.32 | 0.02 | 98.00 | 94.30 | 52.43 |
| Day (60)-Control-9 | 45508958 | 6.83 | 42092236 | 6.31 | 0.03 | 97.07 | 92.38 | 50.95 |
| Day (60)-Control-10 | 41948662 | 6.29 | 41867224 | 6.28 | 0.02 | 98.01 | 94.34 | 50.72 |
| Total | 2774649116 | 416.2 | 2690792188 | 403.64 | / | / | / | / |
| Mean | 46244151.93 | 6.94 | 44846536.5 | 6.73 | 0.028 | 97.65 | 93.65 | 53.67 |
| Standard Deviation | 8524.29 | 0.44 | 3022240.28 | 0.45 | 0.004 | 0.30 | 0.65 | 2.68 |

Note: "Raw Reads": the counts of reads in the raw data; "Raw Bases": the counts of bases in the raw data (raw base = raw reads × 150 bp); "Clean Reads": the counts of reads filtered from the raw data; "Clean Bases": the counts of bases filtered from the raw data (clean base = clean reads × 150 bp); "Error Rate": overall data sequencing error rate; "Q20" and "Q30": the percentage of bases with a Phred > 20 and 30 in the total number of bases, respectively, in the raw bases; "GC Content (%)": the content of GC bases in "clean reads".
